# Cytoplasmic RNA quality control failure engages mTORC1-mediated autoinflammatory disease

**DOI:** 10.1172/JCI146176

**Published:** 2022-01-18

**Authors:** Kun Yang, Jie Han, Mayumi Asada, Jennifer G. Gill, Jason Y. Park, Meghana N. Sathe, Jyothsna Gattineni, Tracey Wright, Christian A. Wysocki, M. Teresa de la Morena, Luis A. Garza, Nan Yan

**Affiliations:** 1 Department of Immunology and; 2 Department of Microbiology, UT Southwestern Medical Center, Dallas, Texas, USA.; 3 Department of Dermatology, Johns Hopkins University School of Medicine, Baltimore, Maryland, USA.; 4 Department of Dermatology,; 5 Department of Pathology and the Eugene McDermott Center for Human Growth and Development,; 6 Department of Pediatrics, and; 7 Department of Internal Medicine, UT Southwestern Medical Center, Dallas, Texas, USA.; 8 Department of Pediatrics, University of Washington and; 9 Seattle Children’s Hospital, Seattle, Washington, USA.

**Keywords:** Autoimmunity, Metabolism, Autoimmune diseases, Genetic diseases, Skin

## Abstract

Inborn errors of nucleic acid metabolism often cause aberrant activation of nucleic acid sensing pathways, leading to autoimmune or autoinflammatory diseases. The SKIV2L RNA exosome is cytoplasmic RNA degradation machinery that was thought to be essential for preventing the self-RNA–mediated interferon (IFN) response. Here, we demonstrate the physiological function of SKIV2L in mammals. We found that *Skiv2l* deficiency in mice disrupted epidermal and T cell homeostasis in a cell-intrinsic manner independently of IFN. *Skiv2l*-deficient mice developed skin inflammation and hair abnormality, which were also observed in a *SKIV2L*-deficient patient. Epidermis-specific deletion of *Skiv2l* caused hyperproliferation of keratinocytes and disrupted epidermal stratification, leading to impaired skin barrier with no appreciable IFN activation. Moreover, *Skiv2l*-deficient T cells were chronically hyperactivated and these T cells attacked lesional skin as well as hair follicles. Mechanistically, SKIV2L loss activated the mTORC1 pathway in both keratinocytes and T cells. Both systemic and topical rapamycin treatment of *Skiv2l*-deficient mice ameliorated epidermal hyperplasia and skin inflammation. Together, we demonstrate that mTORC1, a classical nutrient sensor, also senses cytoplasmic RNA quality control failure and drives autoinflammatory disease. We also propose *SKIV2L*-associated trichohepatoenteric syndrome (THES) as a new mTORopathy for which sirolimus may be a promising therapy.

## Graphical abstract

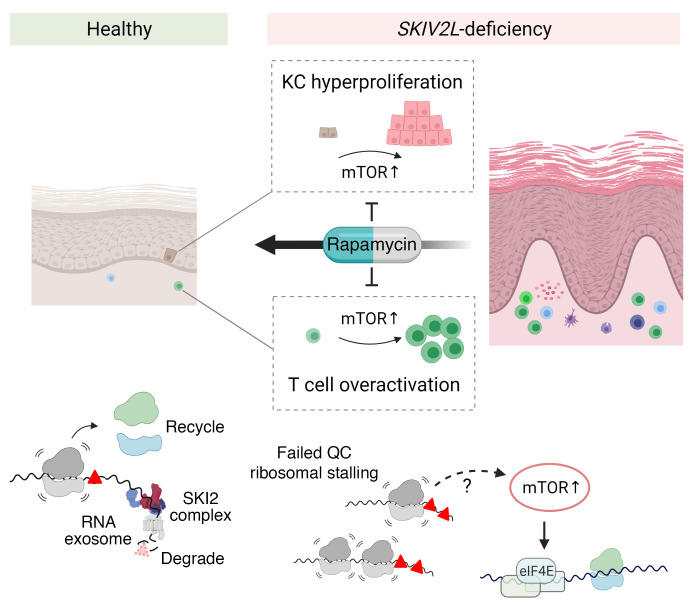

## Introduction

Recognition of microbial nucleic acids by the innate immune system and activation of the type I interferon (IFN) response are fundamental mechanisms of host defense against infection. Inappropriate activation of IFN due to inborn errors of nucleic acid metabolizing enzymes can also drive a variety of autoimmune and autoinflammatory diseases (1). One classical example is *TREX1/DNaseIII* deficiency, which causes accumulation of self-DNA in the cytosol and activation of the DNA-sensing cGAS/STING/IFN pathway. *TREX1* mutants are associated with clinical diseases such as Aicardi-Goutières syndrome and systemic lupus erythematosus (2). Similarly, the accumulation of self-RNA activates the RIG-I–mediated IFN response. The SKIV2L RNA exosome has been shown to degrade self-RNAs in cells undergoing ER stress that produce IRE1-cleaved immunostimulatory self-RNA fragments (3). However, physiological functions of the SKIV2L RNA exosome at homeostasis and in vivo remain unknown.

The RNA exosome is an evolutionarily conserved intracellular 3′ to 5′ RNA degradation complex in eukaryotes, and it is involved in RNA processing, maturation, surveillance, and turnover (4). Mammalian RNA exosomes contain shared catalytic subunits as well as distinct cofactor complexes that bring in different RNA substrates (5). The cofactor for the cytoplasmic RNA exosome is the super-killer (SKI) complex, which contains a Ski2-like RNA helicase (SKIV2L), tetratricopeptide repeat domain 37 (TTC37), and WD repeat domain 61 (WDR61; ref. 6). SKIV2L is an RNA helicase that unwinds RNA substrates and threads them through the RNA exosome for degradation, while TTC37 and WDR61 contribute to the structure and activity of the SKI complex. The SKI complex directly associates with the 80S ribosome and extracts mRNA from stalled ribosomes for degradation as part of ribosome-associated mRNA surveillance (7, 8). Mutations in either the *SKIV2L* or *TTC37* gene are associated with a rare inherited autosomal recessive disorder, trichohepatoenteric syndrome (THES) (OMIM 222470 THES1, 614602 THES2; refs. 9, 10). THES is clinically characterized by intrauterine growth retardation, intractable diarrhea, primary immunodeficiency, liver diseases, and skin and hair abnormalities (11). However, the molecular mechanism of THES remains unknown and no targeted treatment is currently available.

Here, we report that *Skiv2l* deficiency causes skin-specific autoinflammation without triggering innate IFN signaling. Loss of SKIV2L disrupts both epidermal and T cell homeostasis in a cell-intrinsic manner through the mTORC1 pathway, which can be targeted to ameliorate disease pathology associated with *Skiv2l* deficiency.

## Results

### Loss of SKIV2L causes epidermal hyperplasia and impairs skin barrier integrity.

**Figure 1:**
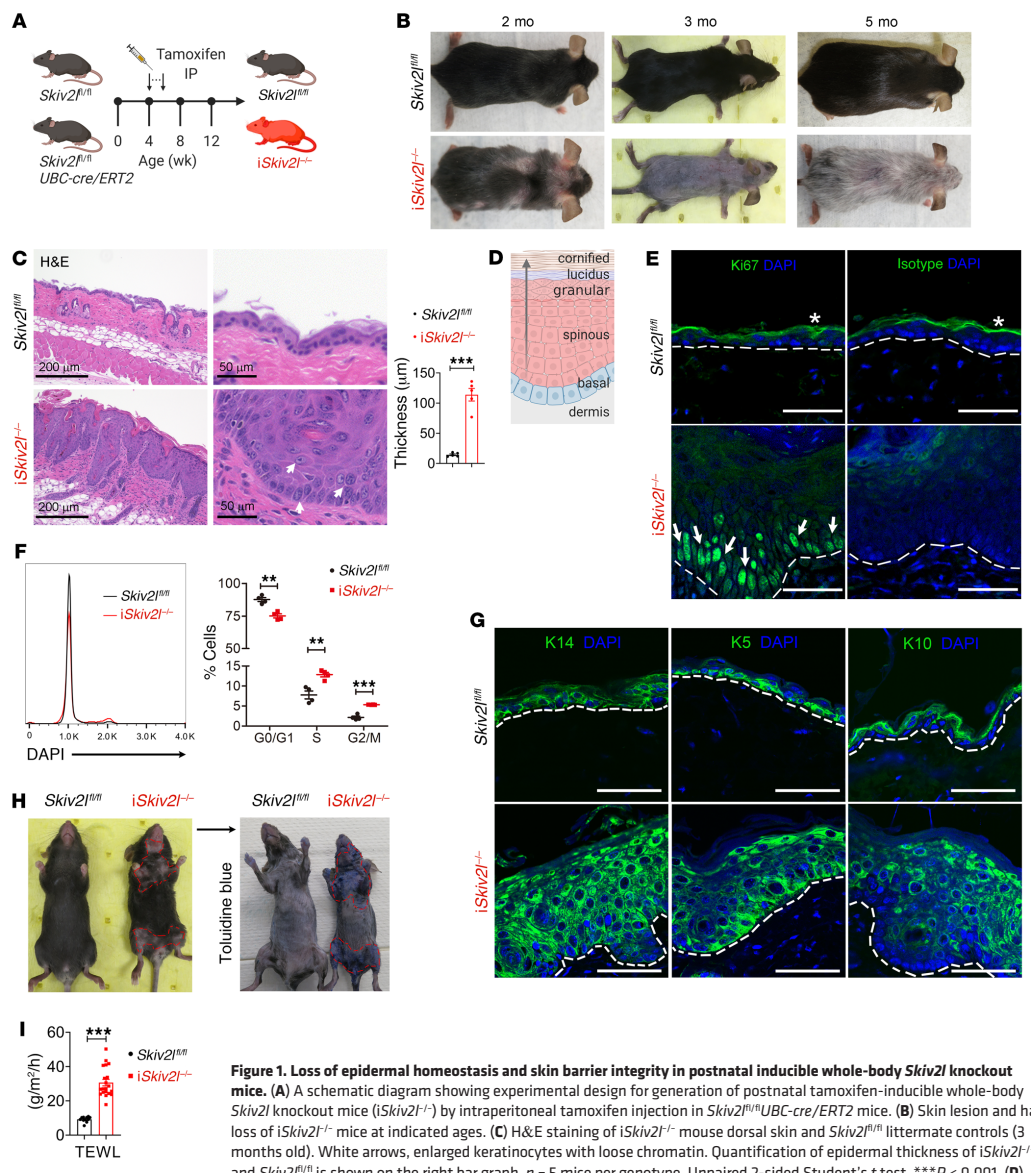
Loss of epidermal homeostasis and skin barrier integrity in postnatal inducible whole-body *Skiv2l* knockout mice. (**A**) A schematic diagram showing experimental design for generation of postnatal tamoxifen-inducible whole-body *Skiv2l* knockout mice (i*Skiv2l*^–/–^) by intraperitoneal tamoxifen injection in *Skiv2l*^fl/fl^*UBC-cre/ERT2* mice. (**B**) Skin lesion and hair loss of i*Skiv2l*^–/–^ mice at indicated ages. (**C**) H&E staining of i*Skiv2l*^–/–^ mouse dorsal skin and *Skiv2l*^fl/fl^ littermate controls (3 months old). White arrows, enlarged keratinocytes with loose chromatin. Quantification of epidermal thickness of i*Skiv2l*^–/–^ and *Skiv2l*^fl/fl^ is shown on the right bar graph. *n =* 5 mice per genotype. Unpaired 2-sided Student’s *t* test, ***P < 0.001. (**D**) A schematic diagram showing epidermal layers. (**E**) Fluorescence immunohistochemistry analysis of proliferation marker Ki67 (green signaling in the nucleus) of i*Skiv2l*^–/–^ mouse dorsal skin and *Skiv2l*^fl/fl^ littermate controls (3 months old). Nuclei were counterstained with DAPI (blue). White arrows, Ki67 positive cells. Asterisk indicates nonspecific staining of stratum corneum of epidermis. Isotype IgG was used as a negative control. Dashed line, epidermal-dermal junction. Scale bar: 50 μm. (**F**) Cell-cycle analysis of keratinocytes isolated from i*Skiv2l*^–/–^ mice and *Skiv2l*^fl/fl^ littermate controls (3 months old). Statistical analysis of cell-cycle distributions are shown on the right. *n =* 4 per genotype. Unpaired 2-sided Student’s *t* test, **P <0.01, ***P < 0.001. (**G**) Fluorescence immunohistochemistry analysis of keratinocyte differentiation markers K14, K5, and K10 in i*Skiv2l*^–/–^ mouse dorsal skin and *Skiv2l*^fl/fl^ littermate controls (3 months old). Nucleus was stained with DAPI (blue). K14, keratin 14; K5, keratin 5; K10, keratin 10. Dashed line, epidermal-dermal junction. Scale bar: 50 μm. (**H**) Representative Toluidine blue staining of depilated i*Skiv2l*^–/–^ mice and *Skiv2l*^fl/fl^ littermate controls. (**I**) TEWL of i*Skiv2l*^–/–^ mice (*n =* 20) and *Skiv2l*^fl/fl^ littermate controls (*n =* 11). Unpaired 2-sided Student’s *t* test, ***P < 0.001.

Germline whole-body deletion of *Skiv2l* resulted in early embryonic lethality before E13.5 (Supplemental Figure 1; supplemental material available online with this article; https://doi.org/10.1172/JCI146176DS1). We therefore generated a *loxP*-flanked *Skiv2l* conditional allele and bred *Skiv2l*^fl/fl^ mice with *UBC-Cre/ERT2* mice to generate *Skiv2l*^fl/fl^*UBC-Cre/ERT2* mice, which allow postnatal whole-body deletion of *Skiv2l* by tamoxifen administration at 4 weeks of age (Figure 1A, we call this i*Skiv2l*^–/–^ throughout). i*Skiv2l*^–/–^ mice have reduced body weight compared with *Skiv2l*^fl/fl^ littermate controls (Supplemental Figure 2A). i*Skiv2l*^–/–^ mice developed phenomenal skin lesions and hair loss 4 weeks after tamoxifen injection (at 8 weeks of age, Figure 1B and Supplemental Figure 2B). Skin redness, scaly plaques, and hair abnormalities were first observed in the skin around the eyes, nose, and neck as early as 2 weeks after induction of *Skiv2l* deletion (Supplemental Figure 2C). By 8 weeks after tamoxifen administration, i*Skiv2l*^–/–^ mice lost most of their body hair, then regrew thin hypopigmented hairs (Figure 1B). Histopathological examination of i*Skiv2l*^–/–^ dorsal skin revealed striking thickening of the epidermis (epidermal hyperplasia) and massive immune infiltrates in the dermis (Figure 1C). We also observed dystrophic anagen hair follicles (HFs) in i*Skiv2l*^–/–^ skin (Supplemental Figure 2D). Surprisingly, we did not observe significant inflammation in duodenum, colon, liver, or kidney of i*Skiv2l*^–/–^ mice, nor any changes in cytokines and chemokines in the serum compared with *Skiv2l*^fl/fl^ littermate controls (Supplemental Figure 2, E and F), suggesting that the inflammation is restricted to the skin even though the *Skiv2l* gene is deleted in the whole body.

The prominent epidermal hyperplasia in the i*Skiv2l*^–/–^ mouse skin prompted us to examine keratinocyte proliferation and differentiation. Normally, keratinocytes in the epidermis continuously differentiate from a proliferative state at the basal layer to a nondividing state at the cornified layer at the top of the skin. This process is tightly regulated to ensure epidermal barrier integrity (Figure 1D). Fluorescence immunohistochemistry analysis of proliferation marker Ki67 showed more proliferating keratinocytes at the basal layer in i*Skiv2l*^–/–^ epidermis compared with that in *Skiv2l*^fl/fl^ controls (Figure 1E). Cell cycle analysis of keratinocytes by flow cytometry also revealed increased mitotic cells in i*Skiv2l*^–/–^ epidermis compared with *Skiv2l*^fl/fl^ control (Figure 1F). Basal keratinocyte markers keratin 14 (K14) and keratin 5 (K5) as well as suprabasal postmitotic marker keratin 10 (K10) all showed massive expansion in i*Skiv2l*^–/–^ mice (Figure 1G), suggesting dysregulation of epidermal stratification. Functionally, i*Skiv2l*^–/–^ mouse skin displayed impaired epidermal permeability as evidenced by the penetration of toluidine blue dye through the lesioned skin (Figure 1H), as well as increased transepidermal water loss (Figure 1I).

### SKIV2L acts cell intrinsically in keratinocytes.

**Figure 2:**
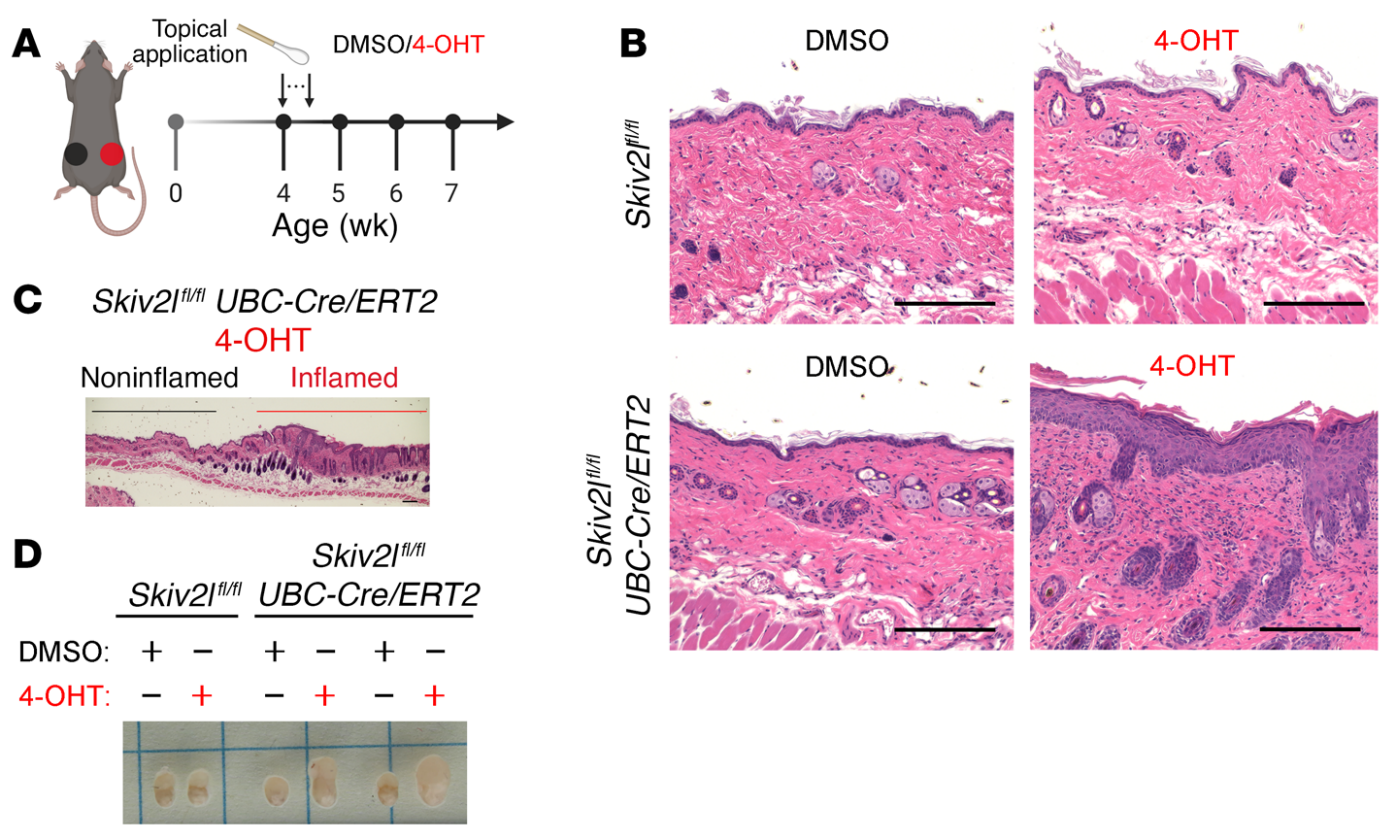
Topical inducible deletion of *Skiv2l* in epidermis results in localized inflammation in the skin. (**A**) A schematic diagram showing experimental design for *Skiv2l* deletion in mouse skin by topical application of 4-OHT at 4 weeks old for 5 consecutive days. DMSO was applied on the contralateral skin as vehicle control. (**B**) H&E staining of 4-OHT- or DMSO-applied skin of *Skiv2l*^fl/fl^*UBC-Cre/ERT2* mice and *Skiv2l*^fl/fl^ littermates 3 weeks after treatment. (**C**) Long section including both 4-OHT–treated and adjacent untreated skin of *Skiv2l*^fl/fl^*UBC-Cre/ERT2* mice. Scale bar: 200 μm. (**D**) Skin-draining inguinal lymph node of 4-OHT– or DMSO-treated skin of *Skiv2l*^fl/fl^*UBC-Cre/ERT2* mice and *Skiv2l*^fl/fl^ littermate controls.

We next analyzed the role of SKIV2L in keratinocytes in 2 additional knockout mouse models. First, we performed local deletion of *Skiv2l* by topically applying 4-hydroxyltamoxifen (4-OHT, the active metabolite of tamoxifen) on the right dorsal flank of the *Skiv2l*^fl/fl^*UBC-Cre/ERT2* mice. We also applied vehicle (DMSO) on the left side of the same mouse as a control (Figure 2A). Topical treatment with 4-OHT induced epidermal thickening and immune infiltration similar to pathologies observed in i*Skiv2l*^–/–^ mice (Figure 2B). No histological abnormality was observed in the vehicle-treated contralateral skin (Figure 2B). In addition, we observed a clear boundary of skin epidermal thickening only in the 4-OHT–treated area but not in the adjacent nontreated area (Figure 2C). We also observed lymphadenopathy of inguinal lymph nodes (LNs) from 4-OHT topical–treated but not vehicle-treated skin (Figure 2D). These data suggest that inducible *Skiv2l* gene deletion locally in the adult mouse skin is sufficient to cause tissue pathology.

**Figure 3:**
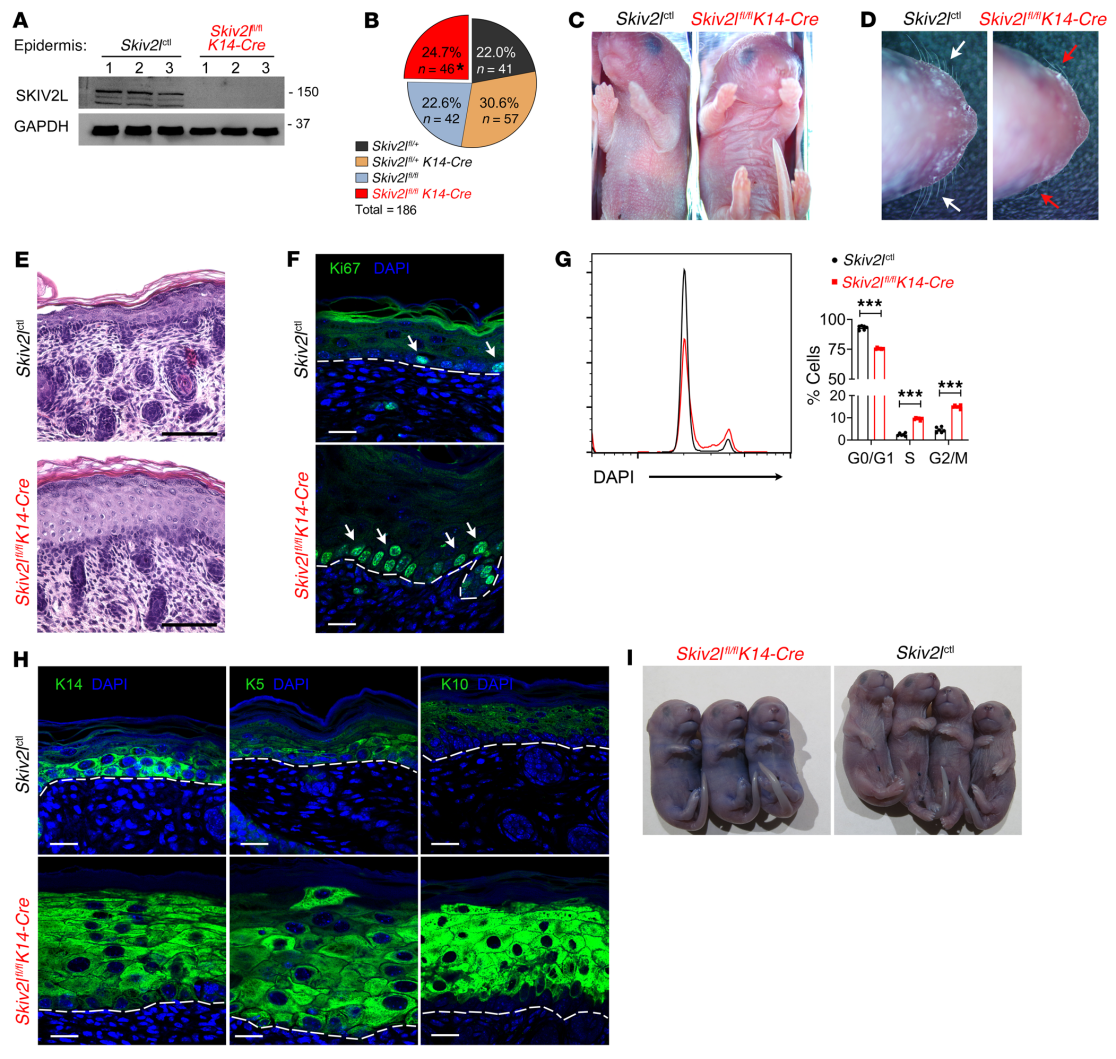
Epidermal hyperproliferation in germline keratinocyte-specific *Skiv2l* knockout mice. (**A**) Western blot analysis of SKIV2L protein in epidermis isolated from *Skiv2l*^ctl^ and *Skiv2l*^fl/fl^*K14-Cre* (germline keratinocyte-specific *Skiv2l* knockout) P0 pups. *n =* 3 per genotype. *Skiv2l*^ctl^ includes *Skiv2l*^fl/fl^, *Skiv2l*^fl/+^, and *Skiv2l*^fl/+^*K14-Cre* and all 3 genotypes showed no phenotypic difference (see Methods). (**B**) A summary of newborn genotypes from genetic crossing of *Skiv2l*^fl/fl^ and *Skiv2l*^fl/+^*K14-Cre* mice. Asterisk indicates all *Skiv2l*^fl/fl^*K14-Cre* newborns died within 24 hours after birth. (**C **and** D**) Skin appearance and whiskers of *Skiv2l*^ctl^ and *Skiv2l*^fl/fl^*K14-Cre* P0 pups. (**E**) H&E staining of *Skiv2l*^ctl^ and *Skiv2l*^fl/fl^*K14-Cre* P0 pup skin. Scale bar: 100 μm. (**F**) Fluorescence immunohistochemistry analysis of proliferation marker Ki67 in *Skiv2l*^ctl^ and *Skiv2l*^fl/fl^*K14-Cre* P0 pup skin tissues. White arrows, Ki67-positive cells. Dashed line, epidermal-dermal junction. Scale bar: 20 μm. (**G**) Cell-cycle analysis of keratinocytes of *Skiv2l*^ctl^ and *Skiv2l*^fl/fl^*K14-Cre* P0 pups. Statistics of cell cycle distributions are shown on the right. *n =* 4 pups per genotype. Unpaired 2-sided Student’s *t* test, ***P < 0.001. (**H**) Fluorescence immunohistochemistry analysis of K14, K5, and K10 in *Skiv2l*^ctl^ and *Skiv2l*^fl/fl^*K14-Cre* P0 pup skin tissues. Dashed line, epidermal-dermal junction. Scale bar, 20 μm. (**I**) Representative Toluidine blue staining of *Skiv2l*^ctl^ and *Skiv2l*^fl/fl^*K14-Cre* P0 pups.

Second, we crossed *Skiv2l*^fl/fl^ with *K14-Cre* mice and generated keratinocyte-specific *Skiv2l* knockout mice (constitutive Cre expression driven by *K14* promoter). Keratinocyte-specific depletion of SKIV2L protein was confirmed by Western blot using *Skiv2l*^fl/fl^*K14-Cre* P0 pup epidermis (Figure 3A and Supplemental Figure 3A). *Skiv2l*^fl/fl^*K14-Cre* mice and littermates were born at expected Mendelian ratio (Figure 3B). However, only *Skiv2l*^fl/fl^*K14-Cre* newborn pups showed abnormal ‘glassy’ skin and died within 24 hours after birth with 100% penetrance (Figure 3C). Although P0 pups have no body hair, we found that *Skiv2l*^fl/fl^*K14-Cre* pups lacked whiskers or developed distorted thin and fine whiskers (Figure 3D), suggesting defective hair morphogenesis. Histological analysis revealed thickening of *Skiv2l*^fl/fl^*K14-Cre* epidermis, particularly in the spinous layer, while no apparent immune infiltration was observed in the dermis (Figure 3E). *Skiv2l*^fl/fl^*K14-Cre* epidermis contained increased proliferating basal keratinocytes compared with controls, as evidenced by positive Ki67 staining and cell-cycle analysis by flow cytometry (Figure 3, F and G). Fluorescence immunohistochemistry analysis further revealed substantial expansion of keratinocytes expressing basal K14 and K5 as well as suprabasal K10 in *Skiv2l*^fl/fl^*K14-Cre* compared with *Skiv2l*^fl/fl^ epidermis (Figure 3H). Toluidine blue exclusion assay revealed impaired barrier integrity of *Skiv2l*^fl/fl^*K14-Cre* mouse skin (Figure 3I). Together, these results suggest that the SKIV2L RNA exosome acts cell-intrinsically in basal keratinocytes to maintain a highly controlled proliferative program during epithelial stratification, and loss of SKIV2L causes uncontrolled expansion of the basal layer and disruption of skin barrier function.

### Skiv2l knockout does not activate IFN signaling in vivo.

We next assessed whether *Skiv2l* knockout activates IFN signaling in vivo. In disease-affected *Skiv2l*^fl/fl^*K14-Cre* P0 pup epidermis, we did not detect any increase in the expression of IFN-stimulated genes (ISGs) compared with littermate controls (Supplemental Figure 3B). We also isolated primary keratinocytes from uninduced *Skiv2l*^fl/fl^*UBC-Cre/ERT2* and *Skiv2l*^fl/fl^ neonates, then induced *Skiv2l* deletion ex vivo by treating cell culture with 4-OHT (Supplemental Figure 4, A and B). We did not observe significant changes in the expression of IFN genes, ISGs, or inflammatory genes, indicating the lack of cell-intrinsic IFN signaling when Skiv2l function is lost in keratinocytes (Supplemental Figure 4C). We further generated myeloid-specific *Skiv2l* knockout mice *Skiv2l*^fl/fl^*LysM-Cre* (Supplemental Figure 5, A–D). *Skiv2l*^fl/fl^*LysM-Cre* mice were born at the expected Mendelian ratio (data not shown) and exhibited normal skin and hair with no signs of skin inflammation or any immunopathology (Supplemental Figure 5, E and F). Cytokines and chemokines in the serum of *Skiv2l*^fl/fl^*LysM-Cre* mice were also indistinguishable from those of *Skiv2l*^fl/fl^ controls (Supplemental Figure 5G). Together, these data suggest that *Skiv2l* knockout does not trigger cell-intrinsic IFN signaling nor myeloid-driven immunopathology in vivo.

### Aberrant activation of the mTOR pathway in Skiv2l-deficient keratinocytes.

**Figure 4:**
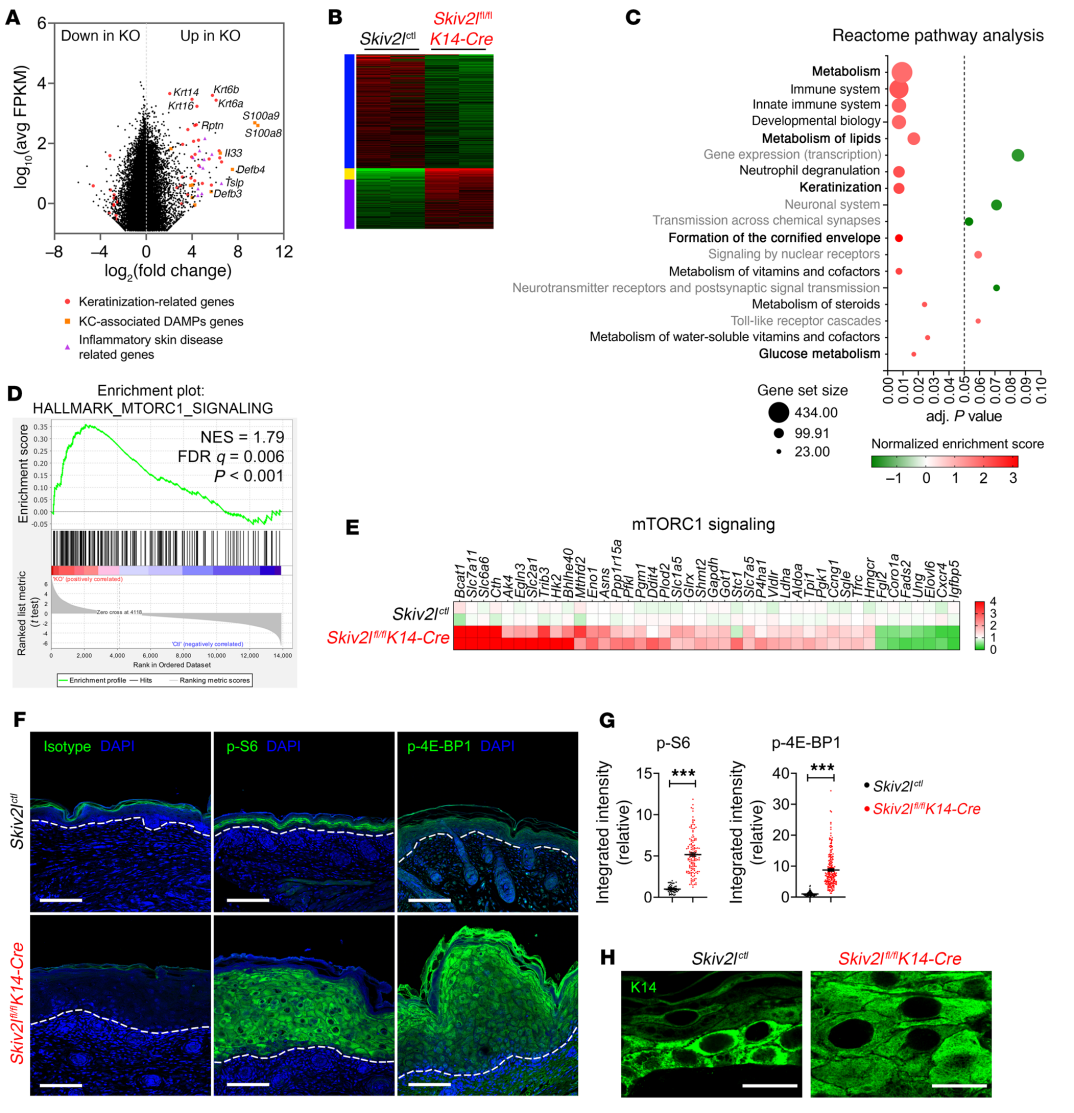
Aberrant activation of the mTORC1 pathway in keratinocytes of germline keratinocyte-specific *Skiv2l* knockout mice. (**A**) MA plot comparing gene expression of *Skiv2l*^ctl^ and *Skiv2l*^fl/fl^*K14-Cre* (germline keratinocyte-specific *Skiv2l* knockout) P0 epidermis (*n =* 2 mice per genotype). Data are shown as average gene expression (log_10_ average FPKM) on the *y* axis and log_2_-fold change (*Skiv2l*^fl/fl^*K14-Cre* [KO] versus *Skiv2l*^ctl^) on the *x* axis, with direction of enrichment as indicated. Red dots, keratinization-related genes; orange squares, keratinocyte-associated DAMPs genes; purple triangles, inflammatory skin disease related genes. (**B**) Heatmap of DEGs of *Skiv2l*^ctl^ versus *Skiv2l*^fl/fl^*K14-Cre*. *n =* 2 mice per genotype. (**C**) Reactome pathway analysis of DEGs of *Skiv2l*^ctl^ versus *Skiv2l*^fl/fl^*K14-Cre*. Dashed line showing adjusted *P* value as 0.05. Pathways without statistical significance (adj. *P* value > 0.05) are shown in gray. Normalized enrichment score (NES) is shown with color bar (red, enriched in *Skiv2l*^fl/fl^*K14-Cre*; green, enriched in *Skiv2l*^ctl^). Circle size indicates number of genes in a gene set. (**D**) GSEA of *Skiv2l*^ctl^ and *Skiv2l*^fl/fl^*K14-Cre* P0 epidermis RNA-seq data set. GSEA plot of hallmark mTORC1 signaling. (**E**) A heatmap showing gene expression of the mTORC1 pathway in *Skiv2l*^ctl^ and *Skiv2l*^fl/fl^*K14-Cre* P0 epidermis. *n =* 2 mice per genotype. (**F **and** G**) Fluorescence immunohistochemistry analysis of p-S6 ribosomal protein (S235/236) and p-4E-BP1 (T37/46) in *Skiv2l*^ctl^ and *Skiv2l*^fl/fl^*K14-Cre* P0 pup skin tissues (**F**). Dashed line, epidermal-dermal junction. Scale bar: 50 μm. Quantification of p-S6 or p-4E-BP1 fluorescence intensity per cell (>50 cells each genotype) is shown in (**G**). Unpaired 2-sided Student’s *t* test, ***P < 0.001. (**H**) Representative images showing enlarged keratinocyte cell size in *Skiv2l*^fl/fl^*K14-Cre* P0 pup skin. Scale bar: 20 μm. The K14 immunofluorescence images of *Skiv2l*^fl/fl^ and i*Skiv2l*^–/–^ epidermis are crops of images shown in Figure 3H.

To explore the underlying mechanism that drives epidermal hyperplasia in *Skiv2l*-deficient mice, we performed whole transcriptome RNA sequencing analysis comparing *Skiv2l*^fl/fl^*K14-Cre* versus *Skiv2l*^ctl^ (includes *Skiv2l*^fl/fl^, *Skiv2l*^fl/+^, and *Skiv2l*^fl/+^*K14-Cre*) P0 mouse epidermis. Differentially expressed genes (DEGs) enriched in *Skiv2l*^fl/fl^*K14-Cre* samples include keratinization-related genes (Krt), keratinocyte damage-associated molecular patterns (DAMPs) genes, and genes associated with inflammatory skin diseases (e.g., psoriasis, atopic dermatitis) (Figure 4A and Supplemental Figure 6, A–C). qRT-PCR analysis of an additional cohort of mice confirmed dysregulated gene expression in *Skiv2l*^fl/fl^ *K14-Cre* epidermis compared with controls (Supplemental Figure 6, D–F). Further pathway analysis revealed enrichment of keratinization and cornification pathways, consistent with the epidermal pathology (Figure 4, B and C).

Interestingly, several metabolic pathways (e.g., lipid and glucose metabolism) were also significantly enriched in *Skiv2l*^fl/fl^*K14-Cre* epidermis compared with controls (Figure 4C). This prompted us to examine the mTOR pathway, which is the master regulator of cellular metabolism. Gene set enrichment analysis (GSEA) using hallmark gene sets revealed enrichment of mTORC1 signaling pathway in *Skiv2l*^fl/fl^*K14-Cre* samples (Figure 4D). The RNA-seq data set showed broad elevation in the expression of the mTORC1 pathway genes in *Skiv2l*^fl/fl^*K14-Cre* dermis compared with controls, which we further confirmed by qRT-PCR analysis using an additional cohort of mice (Figure 4E and Supplemental Figure 6G). We next examined mTORC1 activity by directly measuring phosphorylation of S6 ribosome protein and 4E-BP1, 2 downstream effectors of the mTORC1 pathway. Both p-S6 and p-4E-BP1 were drastically increased in *Skiv2l*^fl/fl^*K14-Cre* and i*Skiv2l*^–/–^ epidermis, quantified as signaling intensity per cell, compared with littermate controls (Figure 4, F and G and Supplemental Figure 7, A and B). These data suggest that *Skiv2l* deficiency activates mTORC1 in keratinocytes.

The SKI complex interacts with the ribosome to extract mRNA from stalled ribosomes for degradation (7, 8). We hypothesized that loss of SKIV2L may impair ribosome recycling on certain mRNAs, which could activate mTORC1 and global protein synthesis. To measure global protein synthesis in vivo, we adapted a pulse-labeling assay using O-propargyl-puromycin (OP-Puro) that can be injected in mice and labels nascent polypeptides in cells. Then, we labeled OP-Puro fluorescently by click chemistry and quantitated protein synthesis in single cells by flow cytometry. i*Skiv2l*^–/–^ keratinocytes showed a significant increase in OP-Puro incorporation, suggesting increased global protein synthesis (Supplemental Figure 7C). We also found that *Skiv2l*-deficient keratinocytes were substantially larger than control cells (Figure 4H and Supplemental Figure 7D), consistent with the notion that mTORC1 controls mammalian cell size by regulating protein translation (12). These data further support mTORC1 activation in *Skiv2l*-deficient cells in vivo.

### Skiv2l deficiency disrupts T cell homeostasis.

Besides epidermal hyperplasia, we also observed immune cell infiltrates, particularly T cells, in i*Skiv2l*^–/–^ mouse dermis and hair follicles (Supplemental Figure 8, A and B). i*Skiv2l*^–/–^ mice exhibited lymphadenopathy and an increase in cellularity of skin-draining lymph nodes (Supplemental Figure 8C). The lymph node in i*Skiv2l*^–/–^ mice exhibits hyperplasia with a larger number of follicles in the paracortex, suggesting an expansion of T cell clusters (Supplemental Figure 8D).

**Figure 5:**
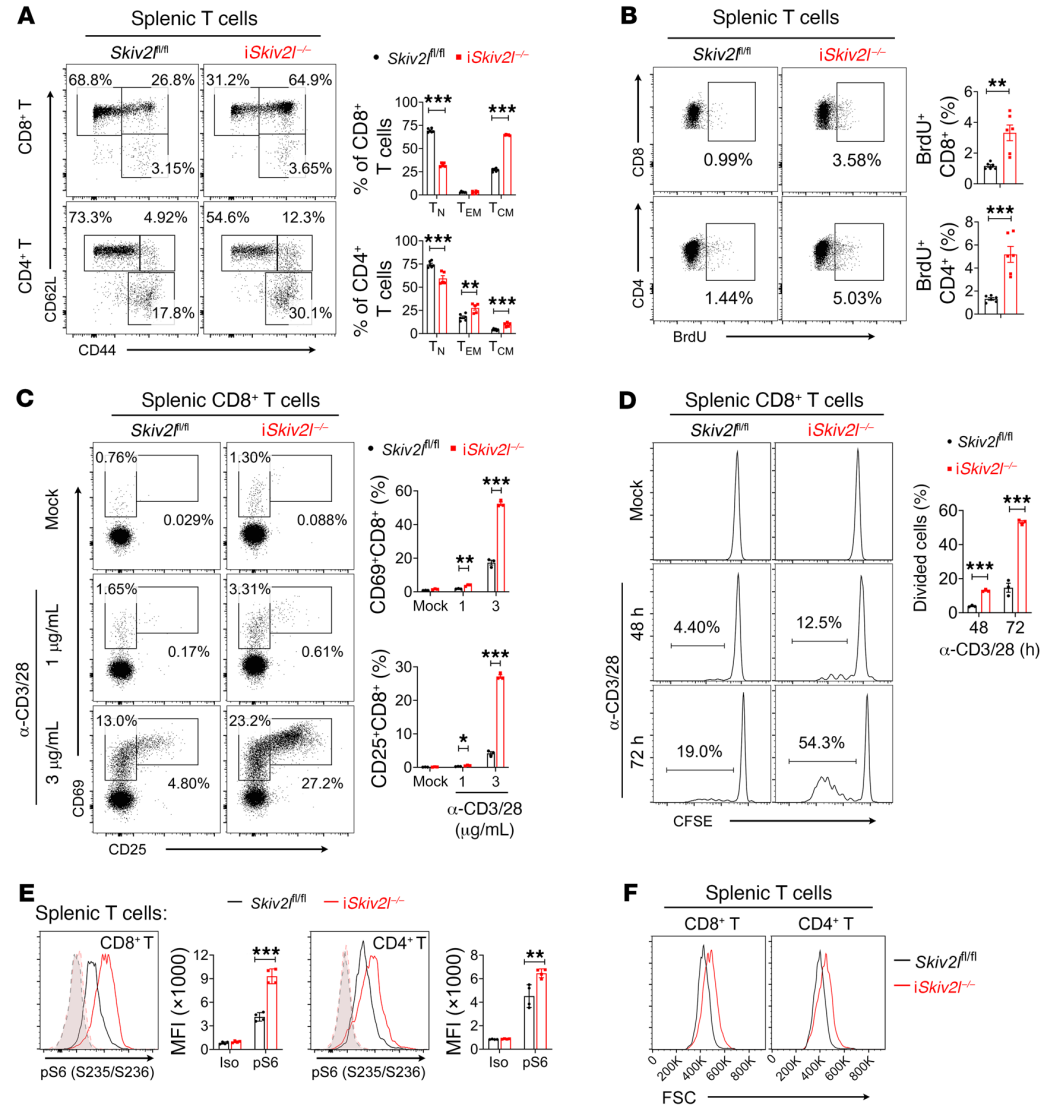
T cell immune homeostasis is disrupted in postnatal whole-body inducible *Skiv2l* knockout mice. (**A**) Flow cytometry analysis of i*Skiv2l*^–/–^ (*n =* 5) and *Skiv2l*^fl/fl^ (*n =* 6) splenic T cells. Numbers adjacent to each gate indicate the percentage of each population. Two-sided Student’s *t* test, **P < 0.01, ***P < 0.001. (**B**) BrdU staining of i*Skiv2l*^–/–^ and *Skiv2l*^fl/fl^ littermates splenocytes 20 hours after injection of BrdU (*n =* 6 mice per genotype). Numbers adjacent to gates (left) in dot plot indicate the percentage of BrdU-positive cells. Two-sided Student’s *t* test, **P < 0.01, ***P < 0.001. (**C**) T cell activation analysis. i*Skiv2l*^–/–^ and *Skiv2l*^fl/fl^ splenic CD8^+^ T cells were stimulated for 16 hours with indicated concentration of anti-CD3 and anti-CD28 antibodies followed by FACS analysis of activation markers CD69 and CD25. Two-sided Student’s *t* test, *P < 0.05, **P < 0.01, ***P < 0.001. (**D**) T cell proliferation analysis by the CFSE dilution assay. i*Skiv2l*^–/–^ and *Skiv2l*^fl/fl^ splenic CD8^+^ T cells were stained with CFSE then stimulated with anti-CD3 and anti-CD28 (3 μg/mL) for indicated times. Two-sided Student’s *t* test, ***P < 0.001. (**E**) Phosphorylation of S6 ribosomal protein (S235/236) in i*Skiv2l*^–/–^ and *Skiv2l*^fl/fl^ splenic T cells. Dashed lines, isotype control (iso). *n =* 4 per genotype. Two-sided Student’s *t* test, **P < 0.01, ***P < 0.001. (**F**) Flow cytometry analysis of T cell size (indicated by FSC). Splenic T cells were isolated from i*Skiv2l*^–/–^ and *Skiv2l*^fl/fl^ mice followed by FACS analysis.

We next examined whether T cell immune homeostasis is perturbed in i*Skiv2l*^–/–^ mice. In the spleen, the number of CD3^+^ T cells and the CD4/CD8 ratio in i*Skiv2l*^–/–^ mice are normal (Supplemental Figure 8, E and F). However, i*Skiv2l*^–/–^ mice showed significantly increased effector memory (CD62L^lo^CD44^hi^) and central memory (CD62L^hi^CD44^hi^) CD4^+^ and CD8^+^ T cells with a corresponding reduction in naive (CD62L^hi^CD44^lo^) T cells in the spleen (Figure 5A and Supplemental Figure 9, A and B), indicating loss of quiescence in *Skiv2l*-deficient T cells. We further evaluated in vivo proliferation of T cells using a bromodeoxyuridine (BrdU) incorporation assay. i*Skiv2l*^–/–^ mice had more BrdU-incorporated T cells than controls under steady-state conditions (Figure 5B). After anti-CD3/CD28 stimulation, expression levels of T cell activation markers CD25 and CD69 were much higher in i*Skiv2l*^–/–^ compared with control T cells (Figure 5C and Supplemental Figure 9C). i*Skiv2l*^–/–^ T cells also proliferated more rapidly and produced more IFN-γ and granzyme B than controls (Figure 5D and Supplemental Figure 9, D–F). To further confirm a cell-intrinsic role of *Skiv2l* deficiency in T cells, we isolated naive CD4^+^ T cells that have not yet encountered antigen and stimulated them ex vivo with anti-CD3/CD28 antibodies (Supplemental Figure 10A). *Skiv2l*-deficient CD4^+^ T cells expressed higher levels of CD25 and CD69 and became more proliferative compared with controls (Supplemental Figure 10, B–D). These results suggest an intrinsic role for SKIV2L in maintaining T cell homeostasis.

We also observed increased p-S6 staining in both CD8^+^ and CD4^+^ T cells of i*Skiv2l*^–/–^ mice compared with controls (Figure 5E). Western blot analysis of sorted splenic T cells further revealed increased phosphorylation of mTORC1 substrates S6K and 4E-BP-1, while phosphorylation of mTORC2 downstream targets FoxO1 and FoxO3a was reduced (Supplemental Figure 11, A–C). i*Skiv2l*^–/–^ CD8^+^ and CD4^+^ T cells are also larger in size than control T cells (Figure 5F). Together, these data suggest that *Skiv2l* deficiency activates mTORC1 and induces T cell hyperactivation.

### mTORC1 inhibitor rapamycin ameliorates iSkiv2l^–/–^ disease pathology.

**Figure 6:**
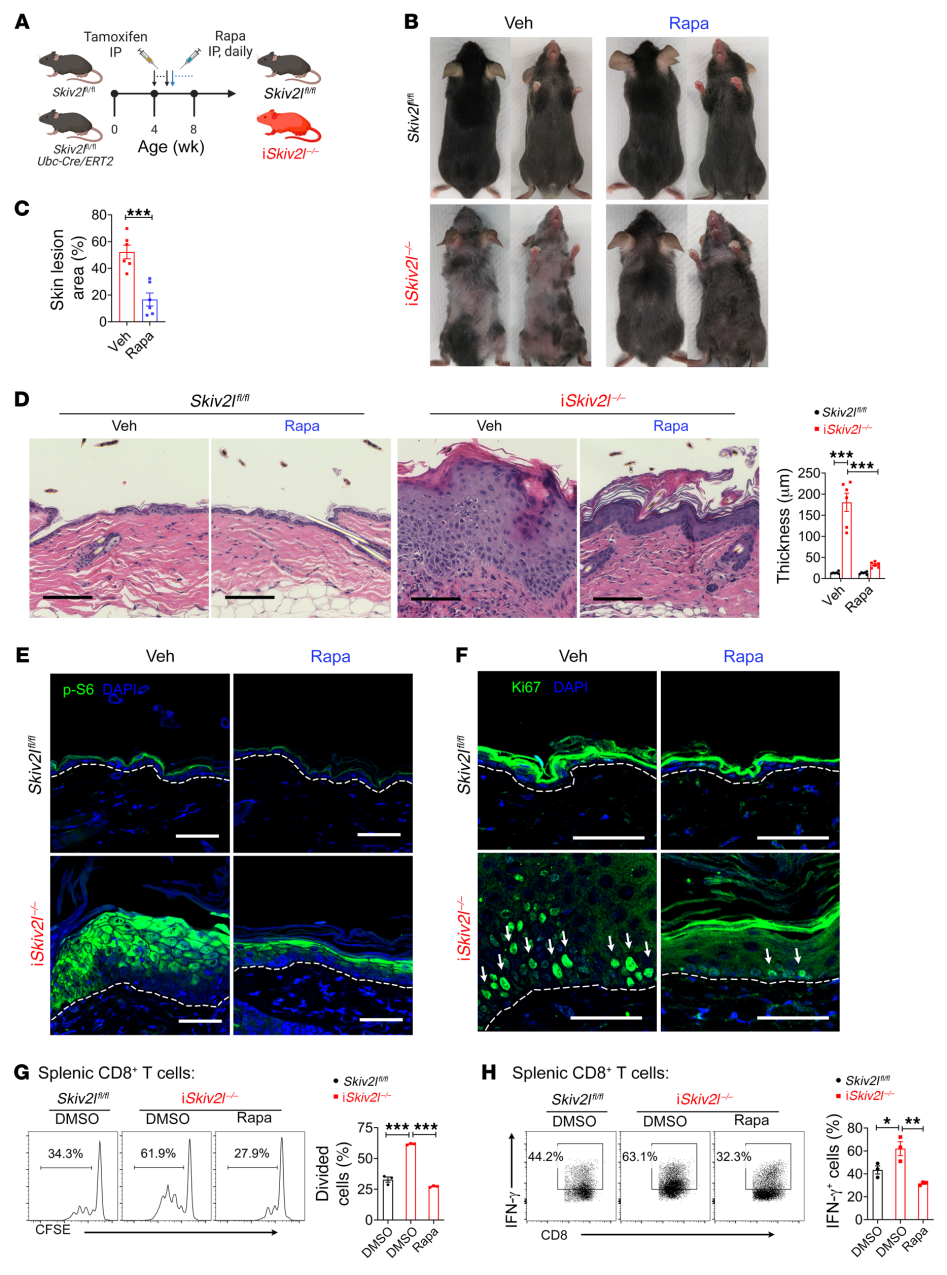
mTORC1 inhibitor rapamycin ameliorates disease pathology in postnatal whole-body inducible *Skiv2l* knockout mice. (**A**) A schematic diagram showing experiment design of rapamycin treatment by intraperitoneal injection in i*Skiv2l*^–/–^ mice and *Skiv2l*^fl/fl^ controls. (**B **and** C**) Representative images of i*Skiv2l*^–/–^ and *Skiv2l*^fl/fl^ mice treated with rapamycin (Rapa, 8 mg/kg body weight, i.p.) or vehicle (veh) for 4 weeks. Quantification of area with lesion on ventral skin is shown on right bar graph (**C**). *n =* 6 per group. Two-sided Student’s *t* test, ***P < 0.001. (**D**) H&E staining of i*Skiv2l*^–/–^ and *Skiv2l*^fl/fl^ mouse dorsal skin after treatment with rapamycin or vehicle (as in **B**). Quantification of epidermal thickness is showing on the right bar graph. *n =* 6 mice per group. Two-way ANOVA with post hoc Tukey’s multiple comparisons test, ***P < 0.001. (**E **and** F**) Fluorescence immunohistochemistry analysis of p-S6 ribosomal protein (S235/236) (**E**) and proliferation marker Ki67 (**F**) of i*Skiv2l*^–/–^ and *Skiv2l*^fl/fl^ mouse dorsal skin after treatment with rapamycin or vehicle (as in **B**). White arrows in **F** denote Ki67-positive cells. Scale bar: 50 μm. (**G**) T cell proliferation analysis by the CFSE dilution assay. i*Skiv2l*^–/–^ and *Skiv2l*^fl/fl^ splenic CD8^+^ T cells were stained with CFSE then stimulated with anti-CD3 and anti-CD28 (3 μg/mL) in the presence of rapamycin or vehicle DMSO. One-way ANOVA with post hoc Tukey’s multiple comparisons test, ***P < 0.001. (**H**) Intracellular IFN-γ staining of i*Skiv2l*^–/–^ and *Skiv2l*^fl/fl^ splenic CD8^+^ T cells stimulated with anti-CD3 and anti-CD28 (3 μg/mL) in the presence of rapamycin or vehicle DMSO. One-way ANOVA with post hoc Tukey’s multiple comparisons test, *P < 0.05, **P < 0.01.

We next assessed whether targeting mTORC1 can reduce disease pathology associated with *Skiv2l* deficiency (Figure 6A). Remarkably, systemic treatment of i*Skiv2l*^–/–^ mice with rapamycin (by intraperitoneal injection) led to significantly reduced skin lesion and hair loss (Figure 6, B and C). Histopathology analysis revealed that rapamycin dramatically ameliorated epidermal hyperplasia in i*Skiv2l*^–/–^ mice (Figure 6D). We also observed reduced p-S6 staining and fewer Ki67^+^ cells within the skin of rapamycin-treated compared with vehicle-treated i*Skiv2l*^–/–^ mice (Figure 6, E and F). Furthermore, rapamycin treatment attenuated hyperproliferation and IFN-γ production of i*Skiv2l*^–/–^ T cells (Figure 6, G and H). Topical rapamycin treatment also reduced epidermal hyperplasia, keratinocyte proliferation and overall skin pathology (Supplemental Figure 12, A–G). These data suggest that mTORC1 is an attractive therapeutic target for *SKIV2L* deficiency.

### mTORC1 activation in a SKIV2L-deficient patient with THES2.

**Figure 7:**
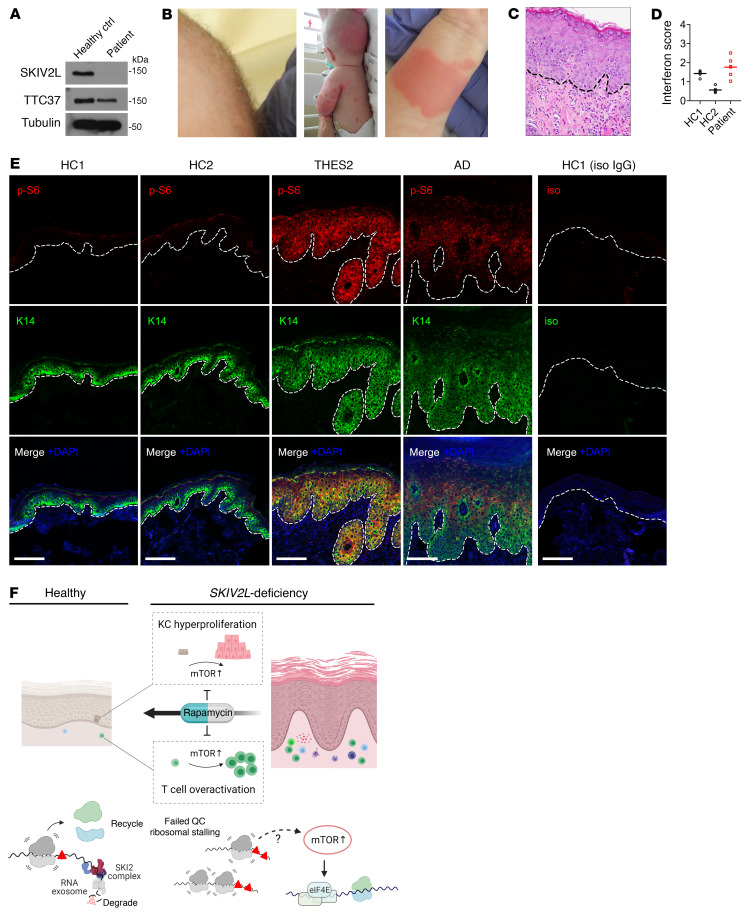
Activation of the mTORC1 pathway in skin tissue from the patient with THES2. (**A**) Western blot analysis of SKIV2L and TTC37 in skin-derived fibroblasts from a patient with THES2 carrying *SKIV2L* mutations and a healthy control. (**B**) Images showing hair abnormalities (so-called woolly hair appearance) and erythematous raised nonpruritic rash of patient with SKIV2L. (**C**) H&E staining of skin biopsy from the patient with THES2 indicates interface dermatitis. Dashed line, epidermal-dermal junction. (**D**) Interferon score of PBMCs from the patient with THES2 and 2 healthy controls. (**E**) Fluorescence immunohistochemistry analysis of p-S6 ribosomal protein (S235/236) and K14 in skin biopsies from healthy controls, the patient with THES2, and an unrelated case of AD. Dashed line, epidermal-dermal junction. Scale bar: 100 μm. (**F**) A schematic model for how *Skiv2l*-deficiency causes mTORC1 activation and disruption to both epidermal and T cell immune homeostasis leading to autoinflammatory skin disease.

We identified a pediatric patient with THES2 who carries 2 pathogenic mutations in each of the *SKIV2L* alleles: c.1452del (p.Val485Cysfs*45) in exon 14 and c.3541-2A>G in intron 27. Both variants are present at extremely low frequencies in the Genome Aggregation Database (gnomAD) and at the time of diagnosis neither variant is present at the Human Gene Mutation Database (HGMD) or ClinVar. SKIV2L protein was undetectable in skin-derived fibroblasts from the patient, and TTC37 protein level was also reduced (Figure 7A). At 8 weeks the patient was admitted to the neonatal intensive care unit (NICU) for failure to thrive (2.1 kg), watery diarrhea, and diagnosis of Group B Streptococcus urinary tract infection, and was found to be viremic with cytomegalovirus. The patient presented with woolly hair appearance indicative of trichorrhexis nodosa (Figure 7B). An erythematous raised nonpruritic rash was noted throughout the body since birth (Figure 7B). Liver studies demonstrated persistent transaminitis. At 5 months, a trial of glucocorticoid therapy was started empirically and resulted in mild skin improvement. Liver biopsies at 7 months were consistent with active hepatitis with mild portal fibrosis and early bridging fibrosis (data not shown). At 17 months, off steroid therapy, the patient was noted to have an increase in blood urea nitrogen (BUN) and creatinine, developed hypertension, and was found to have become positive for p-ANCA and serine protease 3. A kidney biopsy revealed pauci-immune necrotizing and crescentic glomerulonephritis (CKD stage V; Table 1 and Supplemental Figure 13, C and D). There is no history of lupus in either parent of the patient.

Histopathologic analysis of a lesional skin biopsy identified interface dermatitis with lymphocyte infiltration in the dermis (Figure 7C). RNA-seq analysis of PBMCs from the patient with THES2 did not identify elevated ISG expression when compared with healthy controls (Figure 7D and Supplemental Figure 13A). Further immunostaining revealed substantially increased p-S6 in epidermis from the patient with THES2, consistent with activated mTORC1 pathway (Figure 7E). We also noted an interesting difference between THES2 and an unrelated case of atopic dermatitis (AD). p-S6 staining in THES2 is stronger toward the basal layer of the expanded epidermis whereas p-S6 staining in AD is lacking at the basal layer. Further, we observed a moderate increase in MX1 expression in skin tissue from the patient with THES2 (Supplemental Figure 13B). The findings from the patient with THES2 match those from mice and together they suggest that loss of SKIV2L activates mTORC1 signaling, which induces skin autoinflammation (Figure 7F).

## Discussion

Inborn errors in RNA metabolism have been associated with human autoimmune diseases. For instance, Aicardi-Goutières syndrome mutations in RNase H2 (cleaves RNA-DNA hybrids) or in adenosine deaminase acting on RNA 1 (ADAR1) invoke innate immune sensing of self-nucleic acids (DNA or RNA), leading to type I IFN signaling and systemic inflammation (13). Biochemical activities of the cytoplasmic RNA exosome are well-studied in the yeast, but its physiological functions in mammals are not known. Here, using a panel of germline, inducible, whole-body, and tissue-specific gene knockout mouse models, we demonstrate that SKIV2L RNA exosome loss-of-function causes skin-specific autoinflammation by disrupting both epidermal and T cell homeostasis without provoking innate immune response to self-RNA. Tissue-specific deletion of SKIV2L also did not trigger type I IFN response in keratinocytes or myeloid cells. Although MX1 expression is moderately increased in the epidermis of the patient with THES2, it is possible that overactive T cells that infiltrate skin tissue induce ISGs expression in keratinocytes in a paracrine manner. Moreover, we did not observe systemic or multi-organ inflammation when the *Skiv2l* gene was deleted globally in adult mice.

Our in vivo data indicates that the cytoplasmic SKIV2L RNA exosome, under physiological conditions, is unlikely to act as a gatekeeper of self-RNA–mediated innate immune activation. This notion is in line with a recent study investigating substrates of mammalian RNA decay pathways, which reveals that SKIV2L participates widely in surveillance of mRNA with aberrant translation (14). A more comprehensive model could be that the mammalian SKIV2L RNA exosome engages in different functions depending on context or cellular cues. Under physiological conditions, SKIV2L extracts and removes mRNA from stalled ribosomes to avoid activation of mTORC1. Under stress conditions such as viral infection, SKIV2L may expand its role to remove immunogenic host or viral RNA to avoid activation of innate immune sensors.

Our study demonstrates that cytoplasmic RNA quality control failure could activate mTORC1 and cause tissue pathology. The SKI complex directly associates with 80S ribosome and extracts mRNA from stalled ribosome for degradation (7, 8). This mechanism has been proposed to facilitate subsequent dissociation and recycling of stalled ribosomes by the Pelota/Hbs1/ABCE1 complex, though its physiological importance has not been experimentally elucidated. Deletion of *Pelota* leads to global upregulation of protein translation through the mTORC1 pathway and causes epidermal hyperproliferation (15), similar to the *Skiv2l*-deficient skin phenotypes observed in our study. How stalled ribosomes activate the mTORC1 pathway requires further investigation. Nonetheless, these findings and ours collectively demonstrate how seemingly broad cellular defects (RNA quality control, ribosome stalling, and the mTORC1 pathway) can cause remarkably restricted tissue pathology (e.g., skin).

We present a new paradigm of tissue-specific autoinflammation, where metabolically dysregulated T cells (without innate immune activation or priming) are attracted by skin-intrinsic pathology to confer tissue specificity and fuel inflammation. This mechanism is distinct from other skin-specific autoimmune diseases, such as psoriasis and cutaneous lupus erythematosus, which are generally believed to be initiated by aberrant innate immune activation as the primary trigger of pathogenesis (16, 17). Notably, the metabolic perturbation in T cells by *Skiv2l* deficiency seems insufficient to breach global immune tolerance, and we did not observe systemic T cell–mediated autoimmune pathologies that are common in patients with *FOXP3* or *AIRE* mutations (18, 19). While hyper-responsiveness of i*Skiv2l*^–/–^ naive T cells to ex vivo TCR stimulation highly suggest a cell-intrinsic role for SKIV2 in regulating T cell response, it’s worth noting that soluble or secreted factors that naive T cells have engaged with in vivo could also contribute to enhanced T cell response. The patient with THES2 in this study did not develop hemophagocytic syndrome, which is characterized by hyperactivation of lymphocytes and histiocytes, and which is noted in about 60% of patients with THES (20). However, the patient developed ANCA-associated vasculitis, including pauci-immune necrotizing and crescentic glomerulonephritis, which, to our knowledge, is first reported in patients with THES in the present study. Besides autoreactive B cells, expanded effector T cells that are in a persistent activation state are also involved in the pathogenesis (21). The T cell hyperactivation phenotype in our mouse model merits further clinical investigation in patients with THES. The increased mTORC1 activity and decreased mTORC2 activity in *Skiv2l*-deficient T cells are similar to that in systemic lupus erythematosus (SLE) (22–25). Treatment with sirolimus, an mTORC1 inhibitor, corrects the proinflammatory T cell phenotype and improves clinical outcomes in patients with SLE (23, 26). Hyperactivation of the mTORC1 pathway has also been associated with other autoimmune hematological diseases, including autoimmune lymphoproliferative syndrome (ALPS) (27) and idiopathic multicentric Castleman disease (iMCD) (28–30). Aberrant mTOR signaling also contributes to pathogenic lymphoproliferation in those diseases and administration of sirolimus has been shown to induce cellular or clinical benefits in patients (27–32).

Patients with THES develop other clinical symptoms besides skin inflammation, including intractable diarrhea, primary immunodeficiency, and liver disease (11). Some patients with THES2 do not develop characteristic diarrhea, suggesting phenotypic heterogeneity of the disease (33). The whole-body inducible i*Skiv2l^–/–^* mice do not fully replicate all THES phenotypes; this is likely due to incomplete or delayed gene deletion in certain tissues or differences in mouse versus human. Nonetheless, numerous studies have demonstrated a critical role for mTORC1 in intestinal homeostasis (34–36). We demonstrated remarkable therapeutic efficiency of mTOR inhibitor rapamycin in ameliorating skin disease of *Skiv2l*-deficient mice. Therefore, our findings provide a scientifically rationalized and immediately actionable treatment for the THES disease.

## Methods

### Mice.

The C57BL/6N-*A^tm1Brd^* *Skiv2l^tm2a(EUCOMM)Wtsi^*/BayMmucd strain (catalog 037780-UCD) carrying the “knockout-first” *Skiv2l* allele was recovered from the cryo-archive at the Mutant Mouse Resource & Research Centers (https://www.mmrrc.org). The derived heterozygous mice with black appearance were further crossed with B6N.129S4-*Gt(ROSA)26Sor^tm1(FLP1)Dym^*/J mice (B6N.ROSA26:FLPe, Jackson Laboratory, catalog 016226) to delete neomycin selection cassette and generate *Skiv2l*-floxed mice. *Skiv2l*^fl/fl^ mice were further crossed with B6.Cg-*Ndor1^Tg(UBC-cre/ERT2)1Ejb^*/1J (UBC-Cre-ERT2, Jackson Laboratory, catalog 007001), B6.129P2-*Lyz2^tm1(cre)Ifo^*/J (LysM-Cre, Jackson Laboratory, catalog 004781) or B6N.Cg-Tg(KRT14-cre)1Amc/J (K14-Cre, Jackson Laboratory, catalog 018964) to generate tamoxifen-inducible (*Skiv2l*^fl/fl^*UBC-Cre/ERT2*), myeloid-specific (*Skiv2l*^fl/fl^*LysM-Cre*), and epidermis-specific (*Skiv2l*^fl/fl^*K14-Cre*) knockout mice, respectively. As for the crossing of *Skiv2l*^fl/fl^ and *Skiv2l*^fl/+^*K14-Cre* mice, we named the progenies *Skiv2l*^fl/fl^, *Skiv2l*^fl/+^, and *Skiv2l*^fl/+^*K14-Cre* as *Skiv2l*^ctl^ since all 3 genotypes showed no phenotypic difference. The schematic graph of different *Skiv2l* alleles and mouse breeding scheme are illustrated in Supplemental Figure 1A. PCR genotyping of knockout-first and floxed alleles was carried out using the following primers: knockout first allele, forward 5′-CGAATTCTCAGTTGAGCACATAGC-3′, reverse 5′-CCACAACGGGTTCTTCTGTTAG-3′; floxed allele, forward 5′-TACACATGCCTCCATTAGCCTG-3′, reverse 5′-GGCTCATGTCCTGTACACTAATG-3′. PCR genotyping of LysM-Cre, UBC-Cre/ERT2, and K14-Cre mice was carried out following protocols of the Jackson Laboratory. All mice were housed in pathogen-free barrier facilities at UT Southwestern Medical Center.

To generate postnatal whole-body *Skiv2l* knockout mice, tamoxifen (Sigma-Aldrich, catalog T5648) was dissolved in corn oil (20 mg/mL) and injected intraperitoneally into 4-week-old *Skiv2l*^fl/fl^*UBC-Cre/ERT2* and *Skiv2l*^fl/fl^ littermate control mice (75 mg/kg body weight) once daily for 5 consecutive days. To delete *Skiv2l* locally in a small area of skin, *Skiv2l*^fl/fl^*UBC-Cre/ERT2*and *Skiv2l*^fl/fl^ littermate control mice were depilated on both lower flanks. Five microliters of (Z)–4-OHT, Sigma-Aldrich, catalog H7904, dissolved in DMSO to a concentration of 5 μM) or vehicle DMSO was topically applied on right or left lower flank within a circle area (diameter 1 cm) respectively, once daily for 5 consecutive days.

### Human patient study.

The patient included in this study is a female (< 10 years old) born to nonconsanguineous non-Hispanic White parents. Genetic and histopathological analyses of the affected patient were performed with informed consent. Skin fibroblasts were obtained from the patient and expanded in the laboratory. Formalin-fixed paraffin-embedded lesional skin tissue was sectioned for fluorescence immunohistochemistry analysis. Deidentified nondiseased skin samples were obtained from the Tissue Management Shared Resource (TMSR) at UT Southwestern Medical Center. Skin biopsy slides from an unrelated case of AD and an unrelated case of seborrheic keratosis (SK) were kindly provided by Richard C. Wang (UTSW) and used for comparison to the THES2 patient’s skin biopsy.

### Histopathology, immunohistochemistry, and fluorescence immunohistochemistry.

Mouse tissues were fixed in 4% paraformaldehyde and paraffin embedded. H&E staining of mouse tissue was performed at the UT Southwestern Medical Center HistoPathology Core. Immunohistochemistry of mouse skin samples was performed by HistoWiz. H&E staining and immunohistochemistry of the patient’s biopsies were performed and interpreted by a pathologist in Children’s Medical Center Dallas. Fluorescence immunohistochemistry was performed as previously described (37). Briefly, 5 μm paraffin-embedded tissue sections mounted on a slide were deparaffinized and rehydrated. Antigen retrieval was performed in citric acid solution at 121°C for 20 minutes. Slides were blocked with 10% normal goat serum in 1% BSA/PBS for 1 hour at room temperature. The samples were incubated with primary antibodies or isotype control at 4°C overnight. Antibodies used for immunohistochemistry are as follows: rabbit anti-Ki67 (Novus Biologicals, catalog NB500-170, 1:50 dilution), rabbit anti-Keratin 14 (BioLegend, clone Poly19053, catalog 905304, 1:50 dilution), chicken anti-Keratin 14 (BioLegend, clone Poly9060, catalog 906004, 1:50 dilution for costaining with p-S6), rabbit anti-Keratin 5 (BioLegend, clone Poly19055, catalog 905504, 1:50 dilution), chicken anti-Keratin 5 (BioLegend, clone Poly9059, catalog 905903, 1:50 dilution for costaining with p-S6), rabbit anti-Keratin 10 (BioLegend, Poly19054, catalog 905404, 1:50 dilution), phospho-S6 ribosomal protein (Ser235/236) antibody (CST, catalog 2211, rabbit polyclonal, 1:200 dilution), phospho-4E-BP1 (Thr37, Thr46) antibody (Thermo Fisher Scientific, catalog BS-3019R, rabbit polyclonal 1:100 dilution). Slides were washed 3 times and incubated with appropriate conjugated secondary antibodies (goat anti-rabbit IgG (H+L) cross-adsorbed secondary antibody, Alexa Fluor 488, Thermo Fisher Scientific, A-11008, 1:500 dilution; fluoresceinated goat anti-chicken IgY secondary antibody, Aves Labs, F-1005, 1:500 dilution; donkey anti-rabbit IgG (H+L) highly cross-adsorbed secondary antibody, Alexa Fluor 546, Thermo Fisher Scientific, A10040, 1:500 dilution) for 1  hour at room temperature. After nuclei were counterstained with DAPI and tissues were mounted with ProLong Glass Antifade Mountant (Thermo Fisher Scientific, catalog P36983). Immunofluorescence images were acquired using a Zeiss LSM 780 or 880 confocal microscope (Zeiss) at the UTSW Live Cell Imaging Core Facility.

### Flow cytometry.

The antibodies used for flow cytometry are as follows: PerCP-Cy5.5 anti-mouse CD3 (BioLegend, clone 17A2, catalog 100218), APC anti-mouse/human CD45R/B220 (BioLegend, clone RA3-6B2, catalog 103212), FITC anti-mouse/human CD45R/B220 (BioLegend, clone RA3-6B2, catalog 103206), APC anti-mouse CD8a (BioLegend, clone 53-6.7, catalog100712), PE-Cy7 anti-mouse CD8 (BioLegend, clone 53-6.7, catalog 100722), Alex Fluor 700 anti-mouse CD4 (BioLegend, clone GK1.5, catalog 100430), Alex Fluor 488 anti-mouse CD69 (BioLegend, clone H1.2F3, catalog 104516), PE-Cy7 anti-mouse CD25 (BioLegend, clone 3C7, catalog 101916), FITC anti-mouse CD25 (BioLegend, clone 3C7, catalog 101908), FITC anti-mouse CD62L (BioLegend, clone MEL-14, catalog 104406), APC anti-mouse/human CD44 (BioLegend, clone IM7, catalog 103012), APC anti-mouse IFN-γ (BioLegend, clone XMG1.2, catalog 505810), APC Granzyme B (eBioscience, clone NGZB, catalog 17-8898-82), and PE anti-RPS6 Phospho (Ser235/Ser236) (BioLegend, clone A17020B, catalog 608604). For cell surface markers, cells were stained in 1% BSA/PBS with indicated antibodies for 30 minutes at 4°C. For intracellular staining following surface marker staining, cells were fixed and permeabilized using Cytofix/Cytoperm Fixation/Permeabilization Solution Kit (BD Biosciences, catalog 554714), then stained with antibodies for intracellular markers. Dead cells were excluded by using Zombie Aqua Fixable Viability Kit (BioLegend, catalog 423102) during surface marker staining.

For BrdU incorporation assay, BrdU was injected intraperitoneally (2 mg/mouse) and splenocytes were collected 20 hours later for analysis. BrdU staining was performed following BD Pharmingen BrdU Flow Kits (BD Biosciences, catalog 559619) instruction. Phospho-flow cytometry of p-S6 was performed following BD Phosflow Protocols for Mouse Splenocytes or Thymocytes. Data were collected on LSRII flow cytometer (BD Biosciences) or CytoFLEX (Beckman Coulter) and was analyzed using FlowJo software.

### Magnetic-activated cell sorting and T cell in vitro activation.

Magnetic-activated cell sorting (MACS) was performed using autoMACS Pro Separator. The cell isolation kits used were as follows: Pan T Cell Isolation Kit II (Miltenyi Biotec, catalog 130-095-130), Pan B Cell Isolation Kit II, mouse (Miltenyi Biotec, catalog 130-104-443), CD4^+^ T Cell Isolation Kit, mouse (Miltenyi Biotec, catalog 130-104-454), CD8a^+^ T Cell Isolation Kit, mouse (Miltenyi Biotec, catalog 130-104-075), Naive CD4^+^ T Cell Isolation Kit, mouse (Miltenyi Biotec, catalog 130-104-453). Cell labeling and sorting was performed per manufacturer’s instructions. Sorted cells were cultured in plates precoated with anti-CD3 (145-2C11, eBioscience, catalog 16-0031-85) and anti-CD28 (37.51, eBioscience, catalog 16-0281-85) antibodies at the indicated concentration. For CFSE dilution assay, T cells were loaded with CFSE (2.0 μM, Thermo Fisher Scientific, catalog C34570) for 10 minutes at 37°C, then washed with twice complete medium. After culture for indicated time, cells were collected and stained for cell surface or intracellular markers for flow cytometry analysis.

### Flow cytometry analysis of cell cycle and protein synthesis in vivo.

To analyze cell cycle, keratinocytes were isolated from adult mice tail skin and P0 neonate skin using dispase II digestion (Sigma, catalog D4693, 2 mg/mL) at 4°C overnight. Isolated keratinocytes were fixed and permeabilized using Cytofix/Cytoperm Fixation/Permeabilization Solution Kit (BD Biosciences, catalog 554714). Permeabilized keratinocytes were stained with DAPI (10 μg/mL) in Perm/Wash buffer (BD Biosciences) for 30 minutes at 4°C in the dark and analyzed on an LSRII flow cytometer (BD Biosciences) or CytoFLEX (Beckman Coulter) and data were analyzed using FlowJo software. For protein synthesis measurement in vivo, mice were injected intraperitoneally with OP-Puro (Medchem Source, catalog JA-1024) (50 mg/kg body weight, pH 6.4–6.6 in PBS). Mice were euthanized 1 hour later, and dorsal skin was collected. Keratinocytes were isolated as described above. Fluorescence labelling of OP-Puro was performed using Click-iT Plus OPP Alexa Fluor 488 Protein Synthesis Assay Kit per the manufacturer’s instructions (Thermo Fisher Scientific, catalog C10456). Mice injected with PBS were used as control to determine background labelling. Labelled cells were analyzed on LSRII flow cytometer (BD Biosciences) or CytoFLEX (Beckman Coulter). Flow cytometry data were analyzed using FlowJo software.

### RNA sequencing and pathway enrichment analysis.

Total RNA was isolated from the epidermis of P0 pups using RNeasy Mini Kit (Qiagen, catalog 74104). DNA was removed by on-column DNase digestion. RNA integrity was measured by Bioanalyzer (Agilent) at the UT Southwestern Microarray & Immune Phenotyping Core Facility, and samples with RNA Integrity Number greater than 9.0 were used for sequencing. Total RNA sequencing was performed at BGI Americas. Raw sequencing data were processed with Astrocyte RNASeq Analysis Workflows at the UT Southwestern Bioinformatics Core Facility (https://astrocyte.biohpc.swmed.edu/workflow/12/version/324/docs) to generate fragments per kilobase million (FPKM) for each transcript. Protein coding genes with FPKM value greater than 0.5 in at least 1 sample were selected for DEG analysis using iDEP (http://bioinformatics.sdstate.edu/idep/). For MA plot, average gene expression (log_10_-transformed, *y* axis) and *Skiv2l*^fl/fl^*K14-Cre* versus *Skiv2l*^ctl^ fold change (log_2_-transformed, *x* axis) were plotted using GraphPad. Heatmap was generated using iDEP. For pathway enrichment analysis, genes with *Skiv2l*^fl/fl^*K14-Cre* versus *Skiv2l*^ctl^ ratio greater than 1.5 and less than 0.5 were further analyzed using Reactome pathway analysis in iDEP. Gene ontology (GO) Biological Process gene sets (gene set size >20) were used and pathway significant cutoff (false discovery rate) was set as 0.1. Enriched pathway with adjusted *P* values were plotted using GraphPad.

RNA-seq data are available at NCBI GEO (GSE184418). For GSEA, protein-coding transcripts of total RNA sequencing data set were run on GSEA 4.1.0 software (Broad Institute) using H: hallmarks gene sets database with 1000 “gene_set” permutations. The gene set size was specified between 15 and 500, and *t* test was used as the metric for ranking genes.

### RNA isolation and RT-qPCR.

Total RNA was isolated from the epidermis using TRI reagent (Sigma) per the manufacturer’s instructions, and cDNA was synthesized with iScript cDNA Synthesis Kit (Bio-Rad). iTaq Universal SYBR Green Supermix (Bio-Rad) was used to quantify mRNA expression with CFX96 Real-Time PCR Detection System. Gene expression was normalized to internal controls. Primer sequences are shown in Supplemental Table 1.

### Interferon score.

Total RNA was isolated from the patient’s and 2 healthy individuals’ PBMCs using the RNeasy Mini Kit (Qiagen, catalog 74104). RNA integrity was measured by Bioanalyzer (Agilent). mRNAseq strand–specific library was prepared and sequenced on Next Seq SE-75 High Output V2.5 with more than 30 million reads/sample by Genomics Sequencing & Microarray Core at UT Southwestern Medical Center. Primary data analysis was done using CLC-Biosystems Genomic Workbench analysis programs to generate quantitative data for all genes, including RPKM mapped reads values, annotated transcripts, coverage, and chromosomal location. RNA-seq data are available at NCBI GEO (GSE186477). Interferon score was calculated based on the reported methodology (38). Briefly, RPKM for each ISG gene was used to calculate gene expression. The median fold change (relative to mean of 2 healthy controls) of the 6 ISGs (*IFI27, IFI44L, IFIT1, ISG15, RSAD2, SIGLEC1*) was used as the interferon score. Similar methodology was also applied to a broad panel of 67 ISGs.

### Western blot.

Western blots were performed as previously described (39). Briefly, cell lysate was quantified using BCA and an equal amount of proteins were separated on SDS–polyacrylamide gel and transferred to a nitrocellulose membrane. Membranes were blocked with 5% nonfat milk or 3% BSA in 1× TBS-T and incubated with diluted primary antibodies at 4°C overnight per the manufacturers’ instructions. Primary antibodies used were as follows: SKIV2L Rabbit Polyclonal Antibody (Proteintech, catalog 11462-1-AP, 1:500), TTC37 Rabbit Polyclonal Antibody (Proteintech, catalog 24594-1-AP, 1:500), Phospho-mTOR (Ser2448) (D9C2) Rabbit mAb (Cell Signaling, catalog 5536), mTOR (7C10) Rabbit mAb (Cell Signaling, catalog 2983), Phospho-p70 S6 Kinase (Thr389) (108D2) Rabbit mAb (Cell Signaling, catalog 9234), p70 S6 Kinase (49D7) Rabbit mAb (Cell Signaling, catalog 2708), Phospho-S6 Ribosomal Protein (Ser235/236) Antibody (Cell Signaling, catalog 2211), S6 Ribosomal Protein (5G10) Rabbit mAb (Cell Signaling, catalog 2217), Phospho-4E-BP1 (Thr37/46) Antibody (Cell Signaling, catalog 9459), 4E-BP1 Antibody (Cell Signaling, catalog 9452), Phospho-FoxO1 (Ser256) Antibody, (Cell Signaling, catalog 9461), Anti-α-Tubulin antibody, Mouse monoclonal (Sigma, catalog T6199, 1:10,000), GAPDH (14C10) Rabbit mAb (Cell Signaling, catalog 2118), Phospho-FoxO1 (Thr24)/FoxO3a (Thr32) Antibody, (Cell Signaling, catalog 9464), Anti-β-Actin antibody, Mouse monoclonal (Sigma, catalog A1978). Membranes were incubated with HRP-conjugated secondary antibody (Bio-Rad) or IRDye Secondary Antibodies (LI-COR) diluted for 1 hour at room temperature. SuperSignal West Pico Chemiluminescent Substrate (Thermo Fisher Scientific) was used to develop the blots on film or using ChemiDoc Imaging System (Bio-Rad). For blots using IRDye secondary antibodies, blots were scanned using Odyssey DLx Imaging System (LI-COR).

### Cytokine multiplex array.

Mouse serum was collected at indicated ages for cytokine analysis using Bio-Plex Pro Mouse Cytokine 23-plex Assay. The assay was performed at the UT Southwestern Microarray & Immune Phenotyping Core Facility per the manufacturer’s instructions.

### Skin permeability assay.

The skin permeability was evaluated using toluidine blue exclusion assay. In brief, 2-month-old i*Skiv2l*^–/–^ mice and *Skiv2l*^fl/fl^ littermates were shaved after euthanization and rinsed in PBS. The whole body was dehydrated by immersing in 25%, 50%, and 75% methanol/PBS followed by 100% methanol (1 minute each). The mouse skin was then rehydrated with the same series of methanol/PBS solutions (1 minute each), washed in PBS, and stained in 0.0125% toluidine blue O/PBS for 1 minute. The mice were immediately photographed after a brief wash in PBS. Transepidermal water loss (TEWL) of i*Skiv2l*^–/–^ mice and *Skiv2l*^fl/fl^ littermates was measured using Vapometer (Delfin Technologies).

### Rapamycin treatment.

Rapamycin was purchased from LC Laboratory (purity >99%, catalog R-5000). For intraperitoneal treatment, rapamycin was first dissolved in DMSO to 200 mg/mL. Before injection, rapamycin was further diluted in 5% PEG-400/5% Tween-80 to the final concentration of 1.6 mg/mL. After the last dose of tamoxifen injection, mice received intraperitoneal injection of rapamycin (8 mg/kg body weight) or equal volume vehicle daily for 4 weeks. For topical treatment, rapamycin was dissolved in acetone to 1% (wt/vol). Dorsal skin was shaved after the last dose of tamoxifen injection (the day before treatment). Mice received topical applications of 50 μL 1% rapamycin daily on the right dorsal skin and equal volume of acetone on the left dorsal skin for 4 weeks.

### Statistics.

Graphpad Prism was used for illustration of graphs and statistical analysis. Statistical tests performed are indicated in the figure legends. Numerical data were represented as mean ± SEM (error bars). *P* less than 0.05 was considered statistically significant. Schematics and models were created with Biorender.com.

### Study approval.

Mouse studies were carried out in accordance with the NIH’s *Guide for the Care and Use of Laboratory Animals* (National Academies Press, 2011) and the animal protocol was approved by the IACUC at UT Southwestern Medical Center (APN 2017-101968). Human patient studies were reviewed and approved by the IRB at UT Southwestern Medical Center.

## Author contributions

KY performed most of the experiments. JH assisted with mouse breeding and mouse experiments. MA and LAG performed some skin tissue staining and advised on experimental design. JG, JYP, MNS, JGG, TW, CAW, and MTM performed all clinical immunology and pathology analysis. KY and NY designed the project, analyzed the data, and wrote the manuscript with input from all authors.

## Supplementary Material

Supplemental data

## Figures and Tables

**Table 1:**
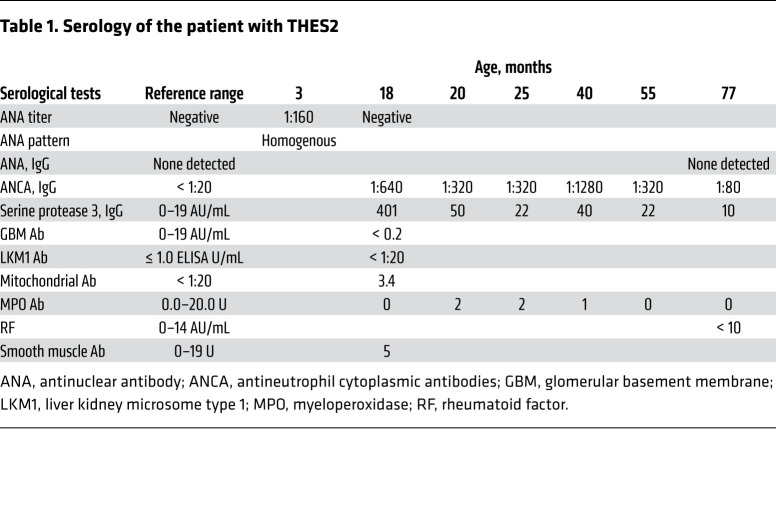
Serology of the patient with THES2

## Version 1 01/18/2022

Electronic publication
