# Supplementary material for: Cytoplasmic RNA quality control failure engages mTORC1-mediated autoinflammatory disease
Source: J Clin Invest. 2022 Jan 18;132(2):e146176. doi: 10.1172/JCI146176 (PMC8759780; doi:10.1172/JCI146176)
Supplement: Supplemental data [file jci-132-146176-s098.pdf]

Supplementary Figures

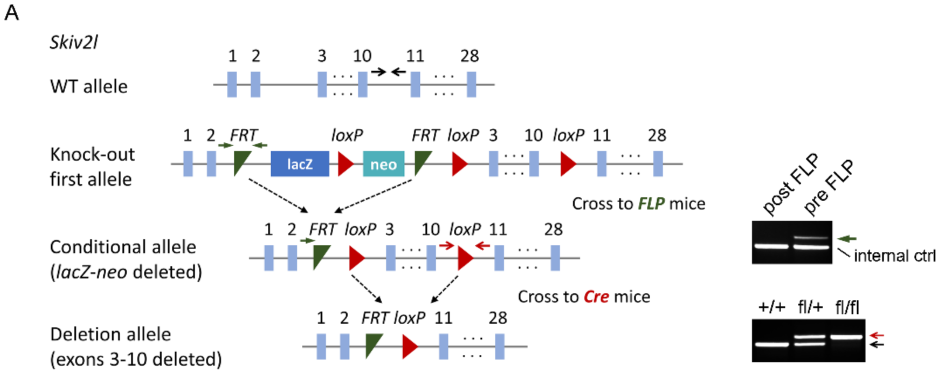

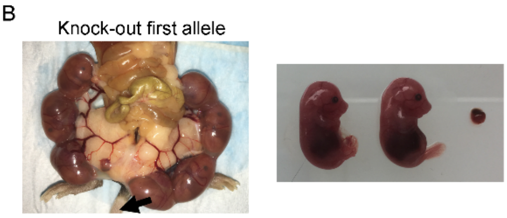

C

Skiv2l	+/+	+/-	-/-
#	58	95	0
%	37.9	62.1	0

Supplemental Figure 1. Generation of *Skiv2l* germline and conditional knockout mice

(**A**) The breeding scheme of *Skiv2l*$^{fl/fl}$ mice and genotyping results of knockout-first and conditional alleles (right graphs).

(**B**) Representative embryos from intercrossing knockout-first-allele heterozygous (*Skiv2l*$^{+/-}$) female breeder mouse at embryonic day E13.5. Arrows indicated absorbed dead embryos. Morphologically normal embryos were either wild-type or heterozygotes.

(**C**) A summary of viable pups from *Skiv2l*$^{+/-}$ intercrossing.

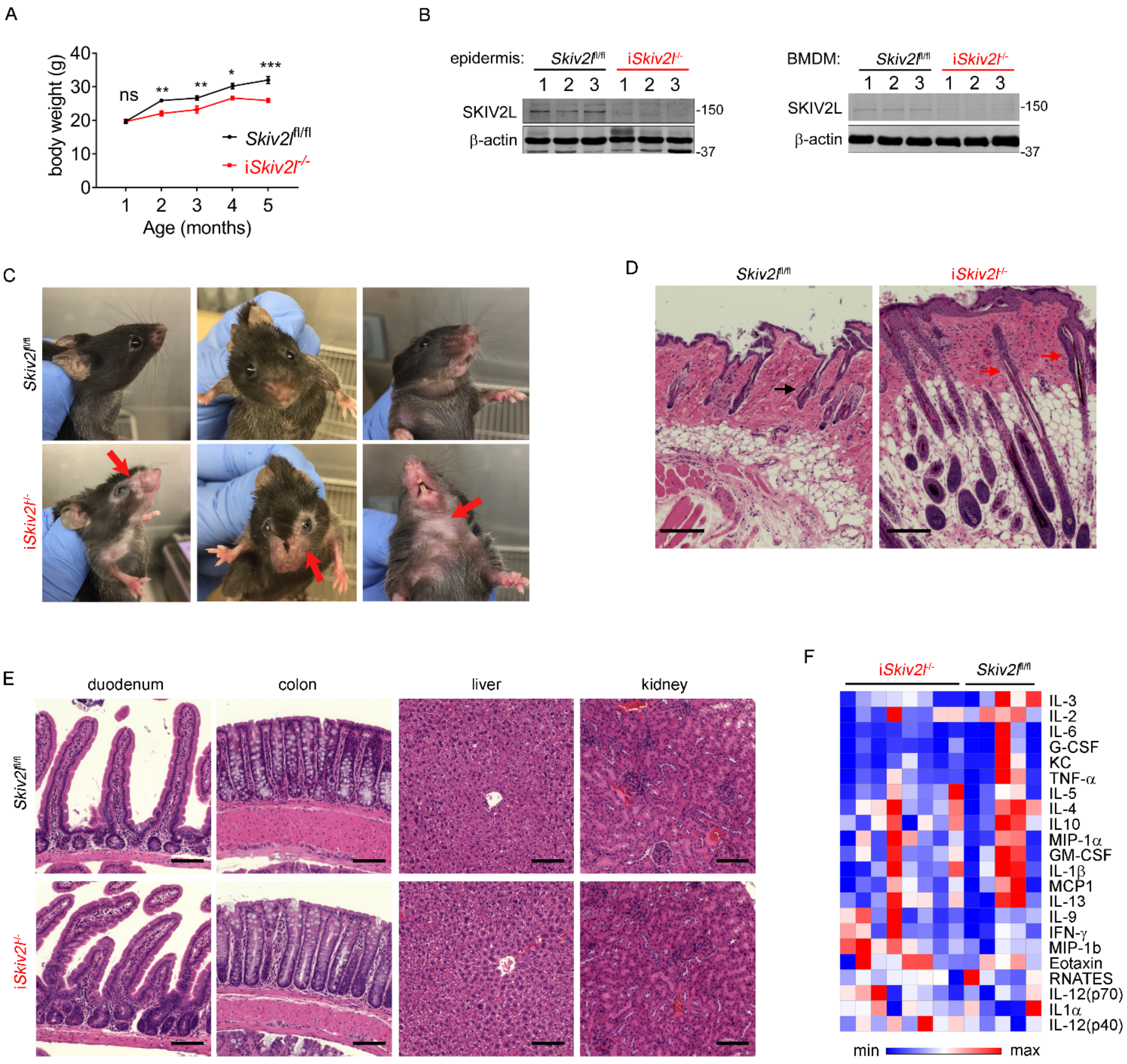

Supplemental Figure 2. Characterization of postnatal inducible whole-body *Skiv2l* knockout mice.

(**A**) Body weight of i*Skiv2l*$^{-/-}$ male mice and *Skiv2l*$^{fl/fl}$ littermate controls. n=4-8 mice per genotype. Unpaired two-sided Student's *t*-test, **P* < 0.05, ***P* < 0.01, ****P* < 0.001. ns, not significant.

(**B**) Western blot analysis of SKIV2L in epidermis and BMDM from i*Skiv2l*$^{-/-}$ mice and *Skiv2l*$^{fl/fl}$ controls. n=3 mice per genotype.

(**C**) Skin lesion, alopecia of i*Skiv2l*$^{-/-}$ mice and *Skiv2l*$^{fl/fl}$ controls at 6 weeks of age.

(**D**) H&E staining analysis of hair follicles (HFs) in dorsal skin of 3-month-old i*Skiv2l*$^{-/-}$ mice and *Skiv2l*$^{fl/fl}$ littermate control. Black arrow, healthy HF without inflammation. Red arrow, dystrophic anagen HF with clustering inflammatory cells. Scale bar, 200 μm.

(**E**) H&E staining of indicated organs of i*Skiv2l*$^{-/-}$ mice and *Skiv2l*$^{fl/fl}$ controls at 3 months of age. Scale bar, 100 μm.

(**F**) Cytokine array analysis of serum of i*Skiv2l*$^{-/-}$ male mice (n=8) and sex-matched *Skiv2l*$^{fl/fl}$ littermates (n=5) at 2 months of age.

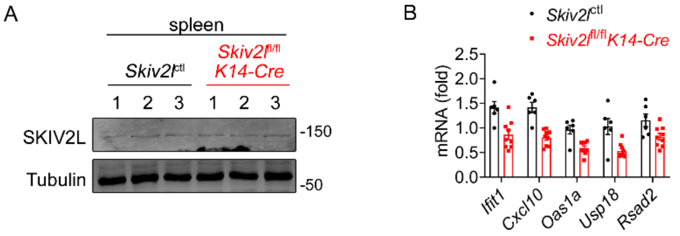

Supplemental Figure 3. Germline keratinocyte-specific *Skiv2l* knockout mice do not show IFN signature in epidermis.

(**A**) Western blot analysis of SKIV2L in spleen from *Skiv2l*^{ctl} and *Skiv2l*$^{fl/fl}$*K14-Cre* P0 pup. n=3 mice per genotype.

(**B**) qPCR analysis of immune gene expression in P0 *Skiv2l*^{ctl} (n=6) and *Skiv2l*$^{fl/fl}$*K14-Cre* (n=9) pup epidermis.

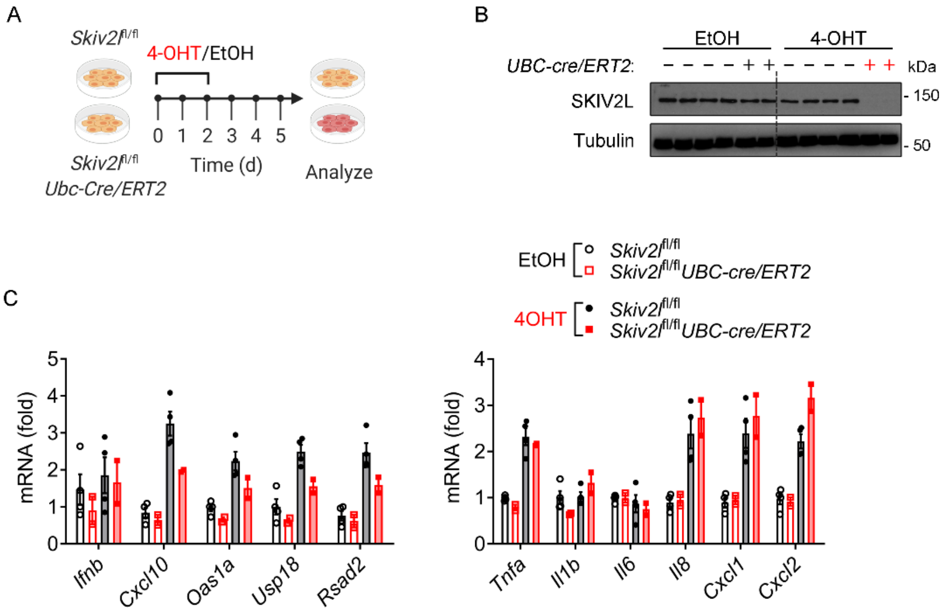

Supplemental Figure 4. Ex vivo deletion of *Skiv2l* in primary keratinocytes does not trigger IFN response.

(**A**) A schematic diagram showing experimental design for ex vitro deletion of *Skiv2l* in primary mouse neonatal keratinocytes. Primary mouse neonatal keratinocytes were isolated from *Skiv2l*$^{fl/fl}$*UBC-Cre/ERT2* P0 pups and *Skiv2l*$^{fl/fl}$ littermates, and treated with 4-hydroxytamoxifen (4-OHT) or vehicle (ethanol, EtOH) for 48 h followed by 72-h culture.

(**B**) Western blot analysis of SKIV2L in primary mouse neonatal keratinocytes from *Skiv2l*$^{fl/fl}$*UBC-Cre/ERT2* P0 pups and *Skiv2l*$^{fl/fl}$ littermates after 4-OHT treatment as in (**A**).

(**C**) qPCR analysis of IFNs, IFN-stimulated genes ISGs, inflammatory cytokine, and chemokine mRNA expression in neonatal keratinocytes of *Skiv2l*$^{fl/fl}$*UBC-Cre/ERT2* mouse and littermate after 4-OHT treatment as in (**A**).

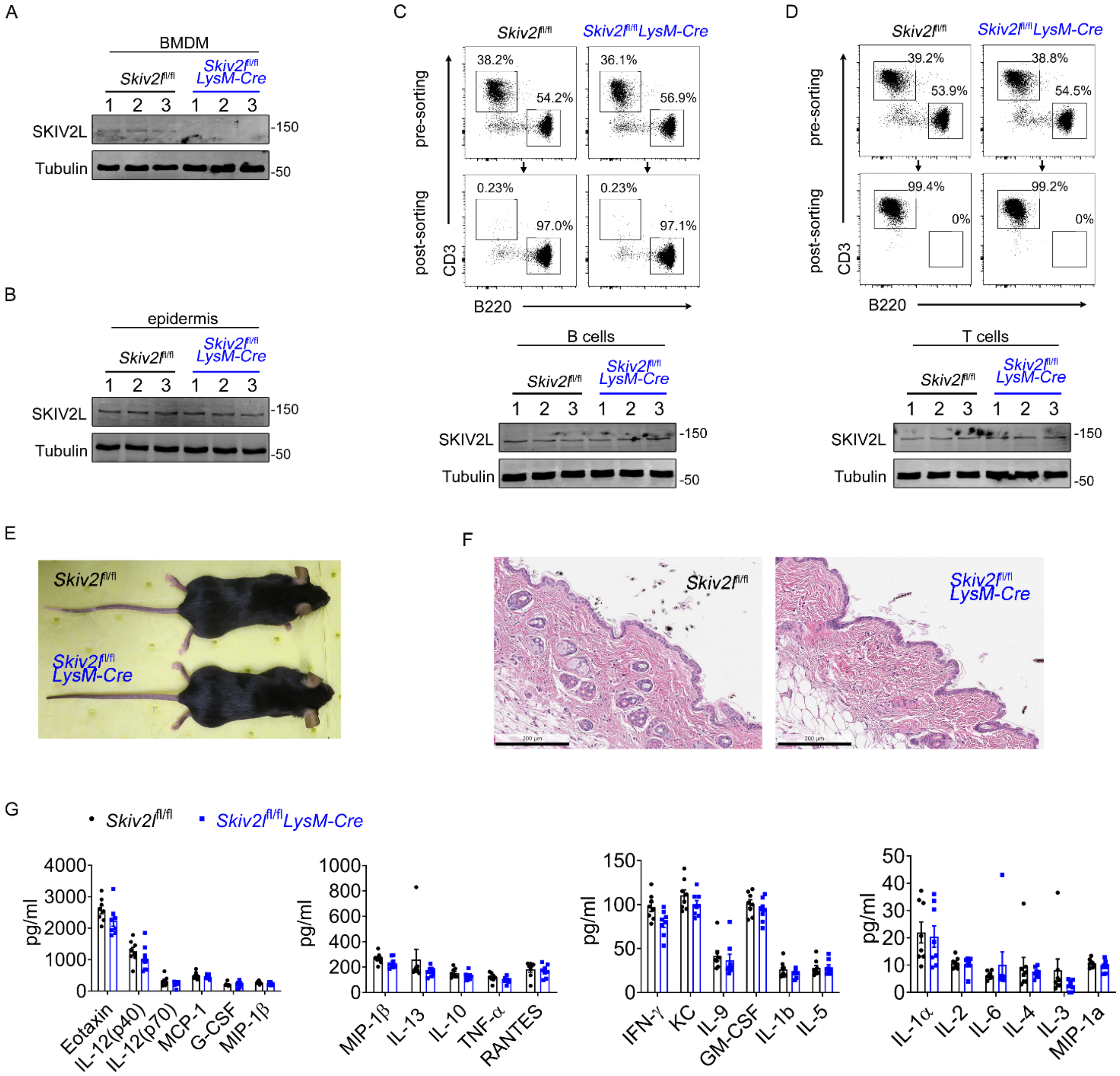

Supplemental Figure 5. Germline myeloid-specific *Skiv2l* knockout does not cause skin disease or systemic inflammation.

(**A, B**) Western blot analysis of SKIV2L in BMDM (**A**) or epidermis (**B**) of *Skiv2l*$^{fl/fl}$*LysM-Cre* mice and *Skiv2l*$^{fl/fl}$ littermate control. n=3 mice per genotype.

(**C, D**) Western blot analysis of SKIV2L in sorted splenic B cells (**C**) or T cells (**D**) of *Skiv2l*$^{fl/fl}$*LysM-Cre* mice and *Skiv2l*$^{fl/fl}$ littermate control. Flow cytometry analysis of sorting purity is shown in upper panels. n=3 mice per genotype.

(**E**) Skin and hair appearance of 7-month-old *Skiv2l*$^{fl/fl}$*LysM-Cre* (germline myeloid-specific *Skiv2l* knockout) mice and *Skiv2l*$^{fl/fl}$ littermates.

(**F**) H&E staining of dorsal skin of 7-month-old *Skiv2l*$^{fl/fl}$*LysM-Cre* mice and *Skiv2l*$^{fl/fl}$ littermates. Scale bar, 200 μm.

(**G**) Serum cytokine analysis of 7-month-old *Skiv2l*$^{fl/fl}$*LysM-Cre* female mice and *Skiv2l*$^{fl/fl}$ littermates. n=9 per genotype.

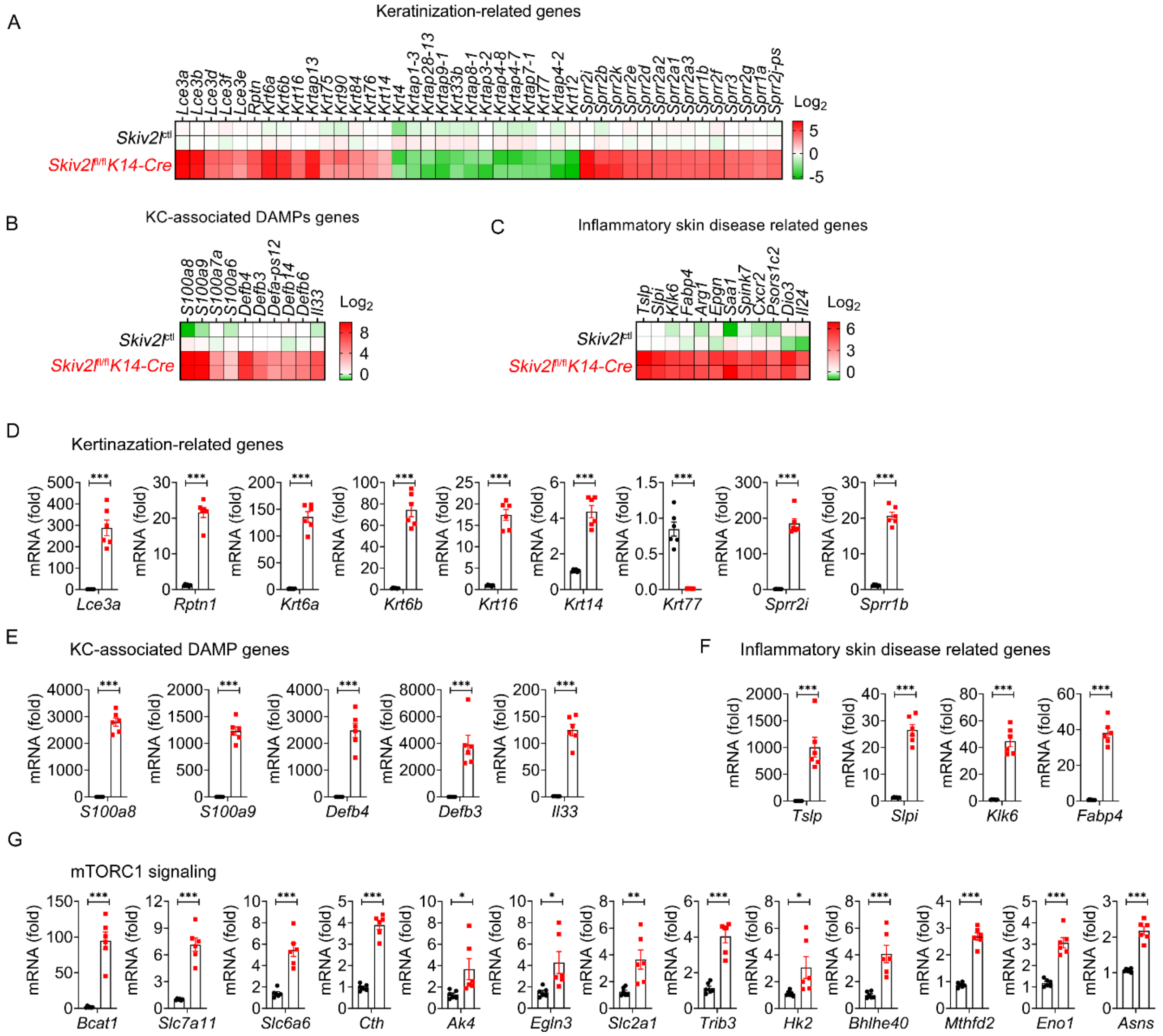

Supplemental Figure 6. Dysregulated pathways in epidermis of germline keratinocyte-specific *Skiv2l* knockout mice.

(**A-C**) A heatmap showing mRNA expression of keratinization-related genes, keratinocyte-associated DAMPs genes and inflammatory skin disease related genes in *Skiv2l*^{ctl} and *Skiv2l*$^{fl/fl}$*K14-Cre* (germline keratinocyte-specific *Skiv2l* knockout) P0 epidermis (n=2 mice per genotype). Relative mRNA level of each gene is shown after normalizing FPKM of each sample to that of the average value of *Skiv2l*^{ctl} samples.

(**D-G**) qRT-PCR analysis of representative gene expression in *Skiv2l*^{ctl} and *Skiv2l*$^{fl/fl}$*K14-Cre* P0 epidermis. n=6 mice per genotype. Two-sided Student's *t*-test, **P*<0.05, ***P*<0.01, ****P* < 0.001.

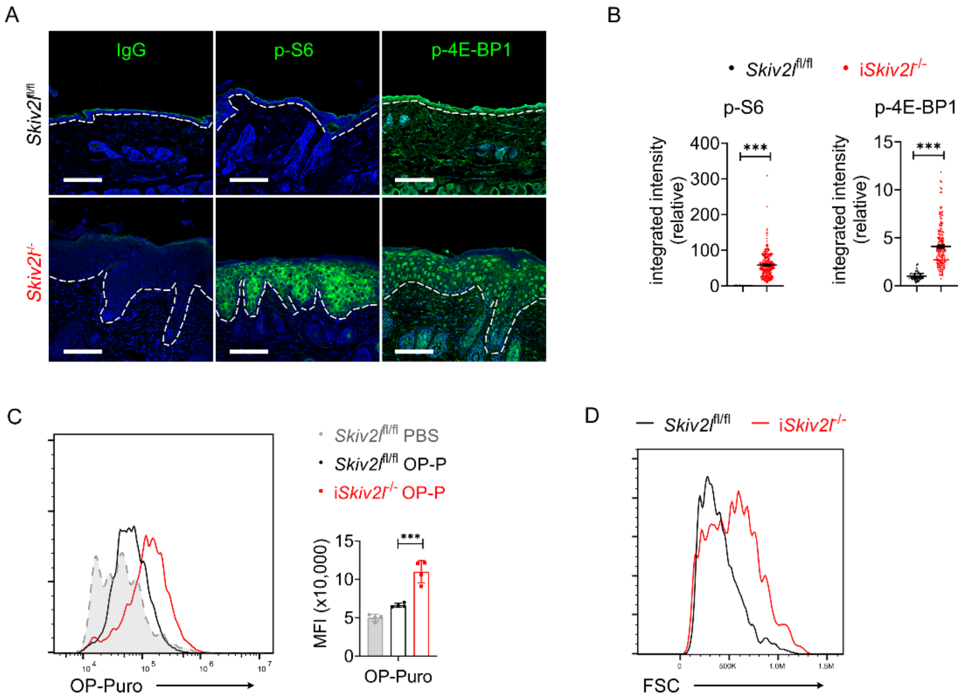

Supplemental Figure 7. Aberrant activation of the mTORC1 pathway in epidermis of postnatal whole-body inducible *Skiv2l* knockout mice.

(**A, B**) Fluorescent immunohistochemistry analysis of phospho-S6 ribosomal protein (S235/236) and phospho-4E-BP1 (T37/46) in dorsal skin of i*Skiv2l*$^{-/-}$ and *Skiv2l*$^{fl/fl}$ mice at 3 months of age (**A**). Dashed line, epidermal-dermal junction. Scale bar, 50 µm. Quantification of pS6 or p4E-BP1 fluorescence intensity per cell (>60 cells each genotype) is shown in (**B**). Two-sided unpaired Student's *t*-test, ***P<0.001.

(**C**) Global protein translation measured by OP-Puro incorporation in keratinocyte of i*Skiv2l*$^{-/-}$ and *Skiv2l*$^{fl/fl}$ mice in vivo. n=4 mice per group. *Skiv2l*$^{fl/fl}$ mice were injected with OP-Puro or PBS and analyzed 1 h after administration. One-way ANOVA with post hoc Tukey's multiple comparison, ***P <0.001.

(**D**) Flow cytometry analysis of keratinocyte cell size (indicated by FSC). Keratinocytes were isolated from i*Skiv2l*$^{-/-}$ and *Skiv2l*$^{fl/fl}$ mice and analyzed by FACS.

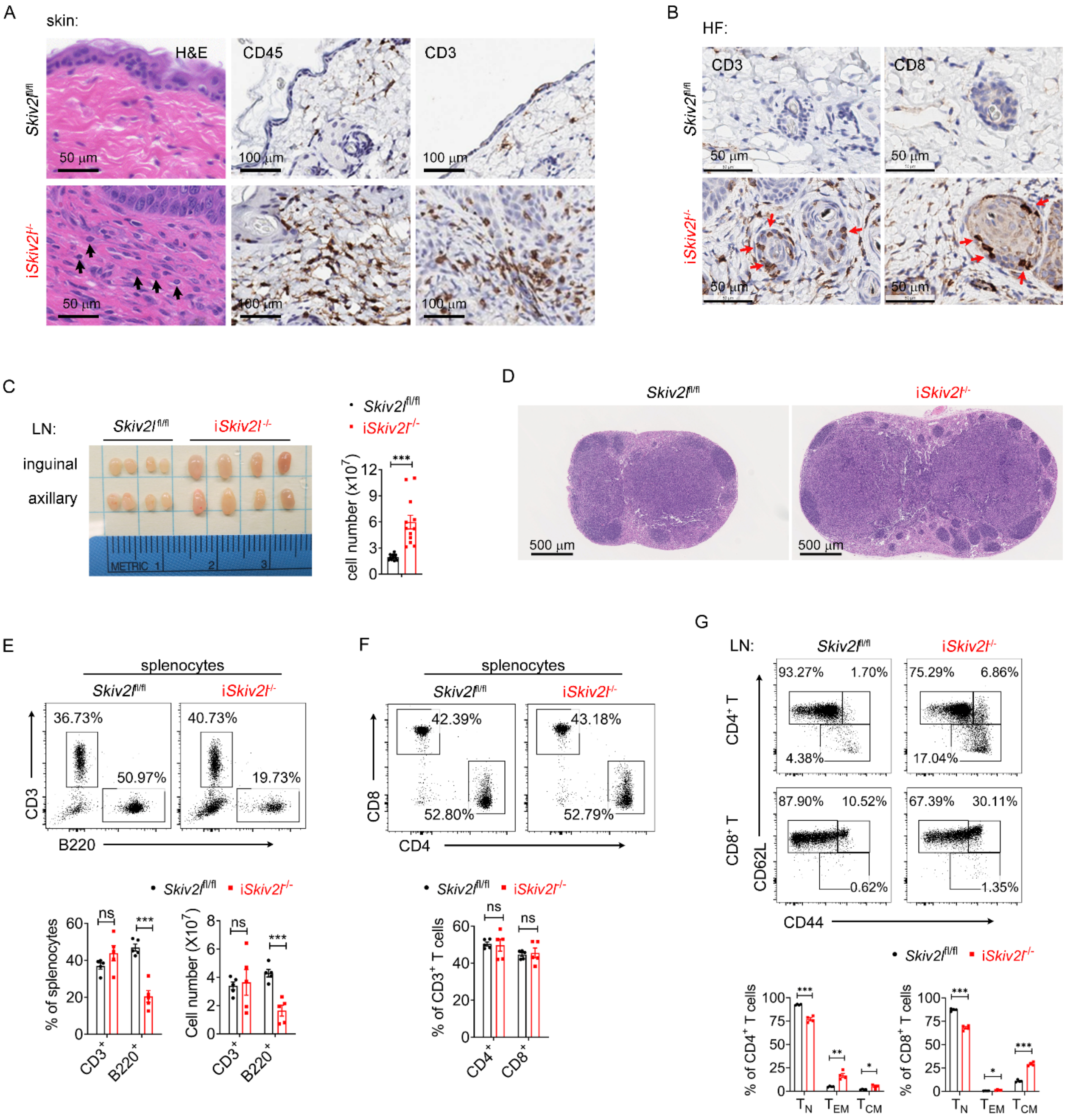

Supplemental Figure 8. Disruption of immune homeostasis in postnatal whole-body inducible *Skiv2l* knockout mice.

(**A**) H&E staining and immunohistochemistry analysis of leukocytes (CD45) and T cells (CD3) in dorsal skin of i*Skiv2l*$^{-/-}$ mice and *Skiv2l*$^{fl/fl}$ littermate control (3-month-old). Black arrows, immune infiltrates in dermis. The H&E staining images of *Skiv2l*$^{fl/fl}$ (top left) and i*Skiv2l*$^{-/-}$ (bottom left) are different crops of images shown in **Figure 1C** (top right and bottom right, respectively).

(**B**) Immunohistochemistry analysis of T cells (CD3, CD8) in hair follicles (HF) of 3-month-old i*Skiv2l*$^{-/-}$ mice and *Skiv2l*$^{fl/fl}$ littermate control. Red arrows, immune infiltrates. Scale bar, 50 µm.

(**C**) Skin-draining inguinal and axillary lymph nodes and their cell numbers (lymph nodes from one side were counted). n=12 mice per genotype. Two-sided Student's *t*-test, ***$P<0.001$.

(**D**) H&E staining of inguinal lymph nodes of i*Skiv2l*$^{-/-}$ mice and *Skiv2l*$^{fl/fl}$ littermate controls (3-month-old).

(**E**) Flow cytometry analysis of $CD3^+$ T cells and $B220^+$ B cells in splenocytes of i*Skiv2l*$^{-/-}$ mice and *Skiv2l*$^{fl/fl}$ controls. n=5 mice per genotype. Two-sided Student's *t*-test, ***P<0.001, ns, not significant.

(**F**) Flow cytometry analysis of $CD4^+$ T cells and $CD8^+$ B cells in spelnocytes (gated on $CD3^+$) of i*Skiv2l*$^{-/-}$ mice and *Skiv2l*$^{fl/fl}$ controls. n=5 mice per genotype. Two-sided Student's *t*-test; ns, not significant.

(**G**) Flow cytometry analysis of i*Skiv2l*$^{-/-}$ and *Skiv2l*$^{fl/fl}$ inguinal lymph node T cells. Numbers adjacent to each gate indicate the percentage of each population. n=3 mice per genotype. Two-sided Student's *t*-test, *P<0.05, **P<0.01, ***P<0.001.

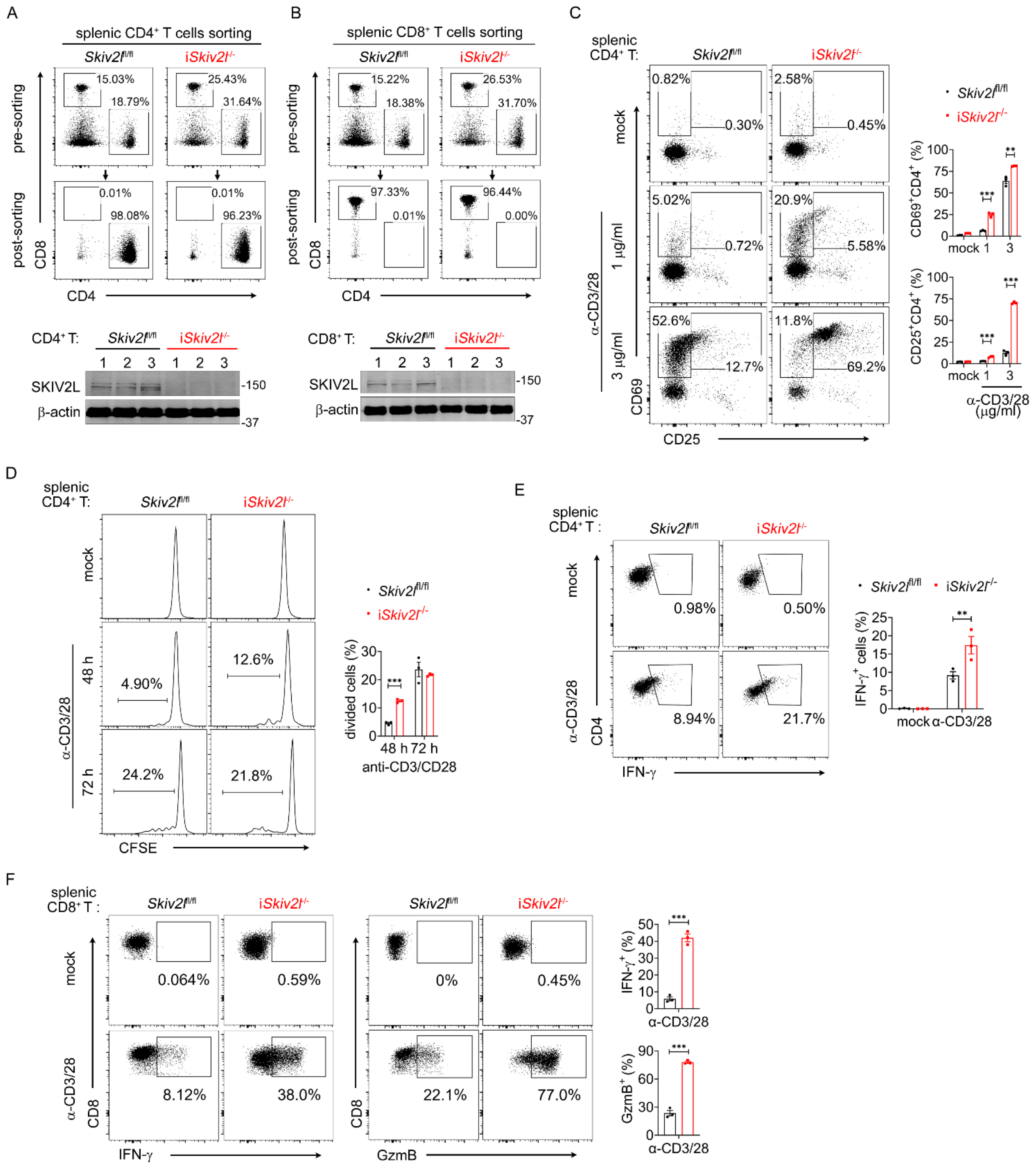

Supplemental Figure 9. T-cell hyperactivation in postnatal inducible whole-body *Skiv2l* knockout mice. (**A, B**) Western blot analysis of SKIV2L in sorted splenic CD4$^+$ (**A**) or CD8$^+$ T cells (**B**) of i*Skiv2l*$^{-/-}$ mice and *Skiv2l*$^{fl/fl}$ littermate controls. Flow cytometry analysis of sorting purity is shown in upper panels. n=3 mice per genotype.

(**C**) Expression of activation markers of i*Skiv2l*$^{-/-}$ and *Skiv2l*$^{fl/fl}$ splenic CD4$^+$ T cells stimulated with indicated concentration of anti-CD3 and anti-CD28 antibodies for 16 h. n=3 per genotype. Two-sided Student's *t*-test, $^{**}P<0.01$, $^{***}P<0.001$.

(**D**) T cell proliferation analysis by CFSE dilution assay. i*Skiv2l*$^{-/-}$ and *Skiv2l*$^{fl/fl}$ splenic $CD4^+$ T cells were stained with CFSE then stimulated with anti-CD3 and anti-CD28 (3 μg/ml) for indicated times. n=3 per genotype. Two-sided Student's *t*-test, ***P<0.001.

(**E, F**) Intracellular IFN-γ or granzyme B (GzmB) staining of i*Skiv2l*$^{-/-}$ and *Skiv2l*$^{fl/fl}$ splenic $CD4^+$ T (**E**) and $CD8^+$ T cells (**F**) after stimulation with anti-CD3 and anti-CD28 (3 μg/ml). Two-sided Student's *t*-test, **P<0.01, ***P<0.001.

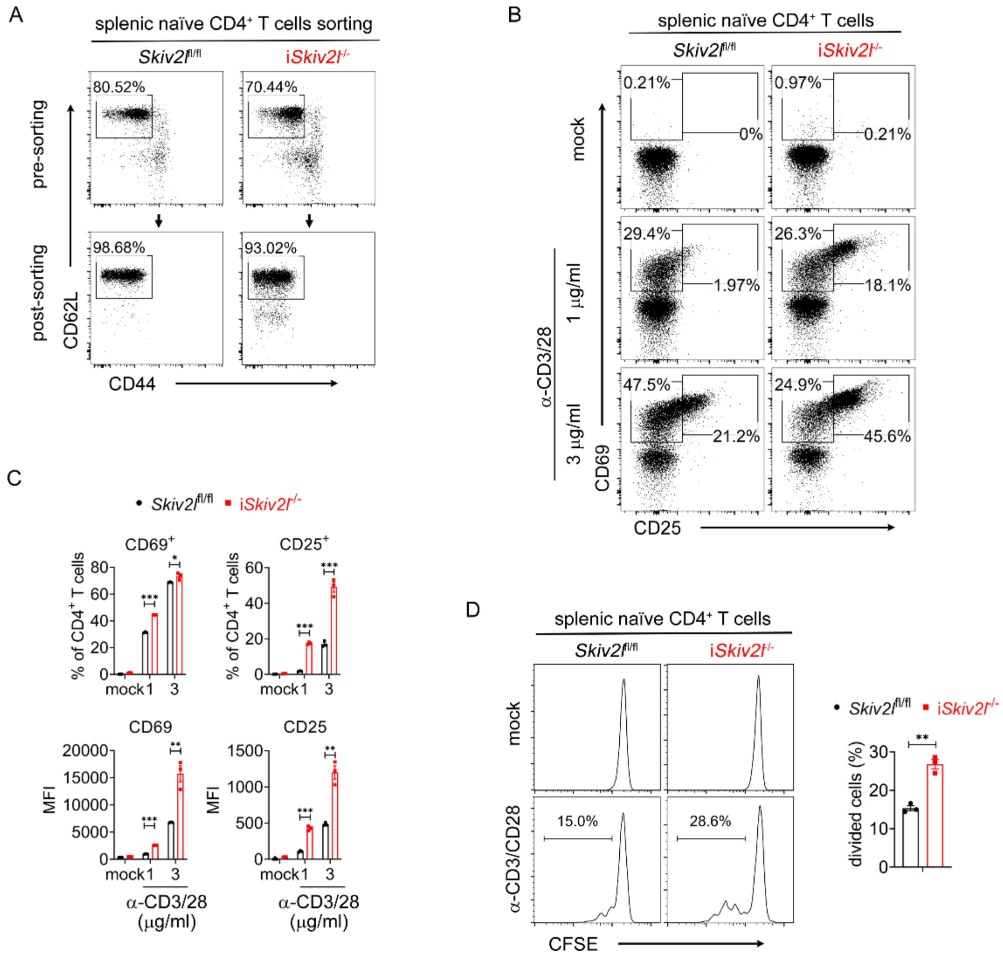

Supplemental Figure 10. Hyperactive response of *Skiv2l*-deficient naïve CD4$^+$ T cells.

(**A**) Flow cytometry analysis of naïve CD4$^+$ T cells purity after sorting from i*Skiv2l*$^{-/-}$ and *Skiv2l*$^{fl/fl}$ splenocytes.

(**B, C**) Expression of activation markers CD69 and CD25 of i*Skiv2l*$^{-/-}$ and *Skiv2l*$^{fl/fl}$ naïve CD4$^+$ T cells stimulated with indicated concentration of anti-CD3 and anti-CD28 antibodies for 16 h. n=3 per genotype. Two-sided Student's *t*-test, **P*<0.05, ***P*<0.01, ****P*<0.001.

(**D**) T cell proliferation analysis by CFSE dilution assay. i*Skiv2l*$^{-/-}$ and *Skiv2l*$^{fl/fl}$ naïve CD4$^+$ T cells were stained with CFSE then stimulated with anti-CD3 and anti-CD28 (3 µg/ml) for 72 h. n=3 per genotype. Two-sided Student's *t*-test, ***P*<0.01.

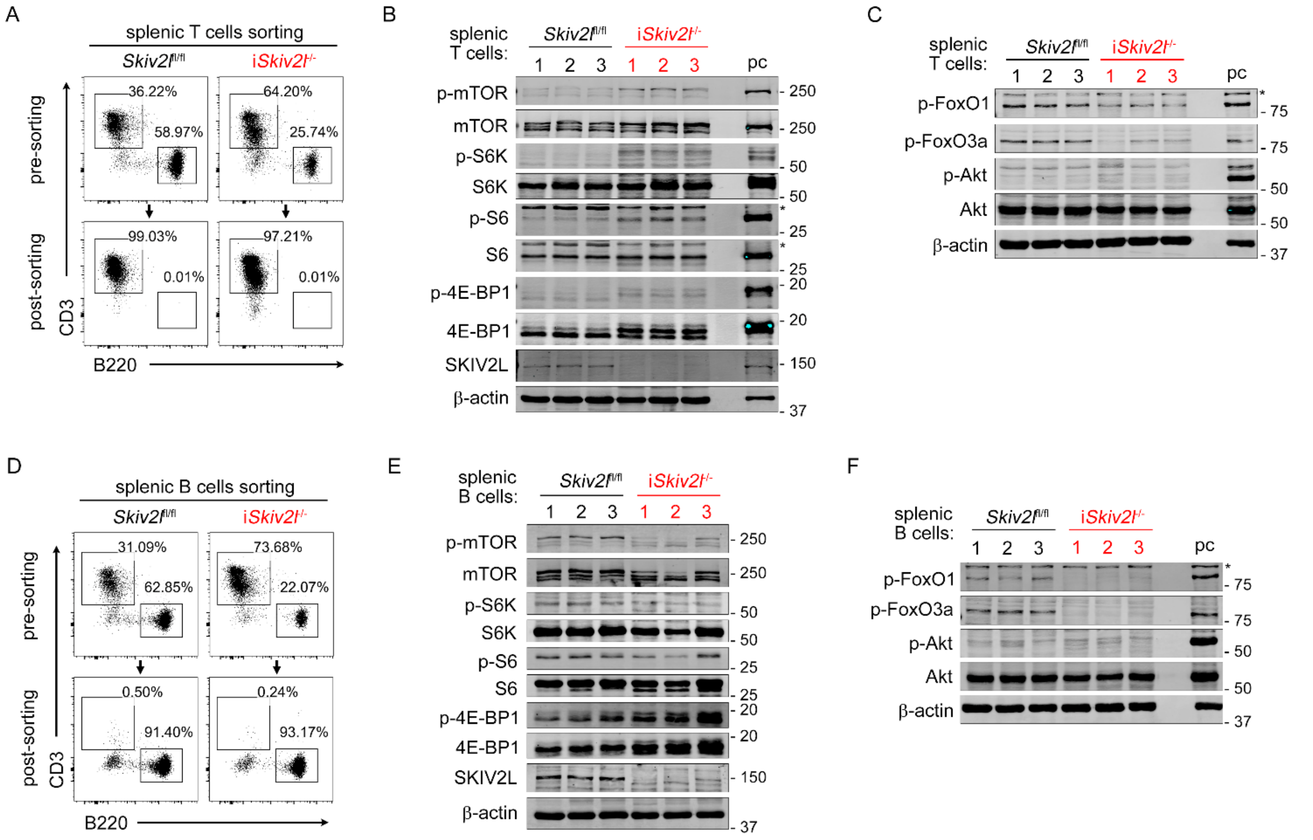

Supplemental Figure 11. Activation of the mTORC1 pathway in T cells of postnatal inducible whole-body *Skiv2l* knockout mice.

(**A**) Flow cytometry analysis of T cells purity after sorting of i*Skiv2l*$^{-/-}$ and *Skiv2l*$^{fl/fl}$ splenocytes.

(**B, C**) Western blot analysis of mTORC1 (**B**) and mTORC2 (**C**) pathway in sorted splenic T cells of i*Skiv2l*$^{-/-}$ mice and *Skiv2l*$^{fl/fl}$ controls. n=3 mice per genotype. Whole cell lysate of B16 melanoma cells was used as positive control (pc). *, non-specific band.

(**D**) Flow cytometry analysis of B cells purity after sorting of i*Skiv2l*$^{-/-}$ and *Skiv2l*$^{fl/fl}$ splenocytes.

(**E, F**) Western blot analysis of mTORC1 (**E**) and mTORC2 (**F**) pathway in sorted splenic B cells of i*Skiv2l*$^{-/-}$ mice and *Skiv2l*$^{fl/fl}$ controls. n=3 mice per genotype. Whole cell lysate for B16 melanoma cells was used as positive control (pc). *, non-specific band.

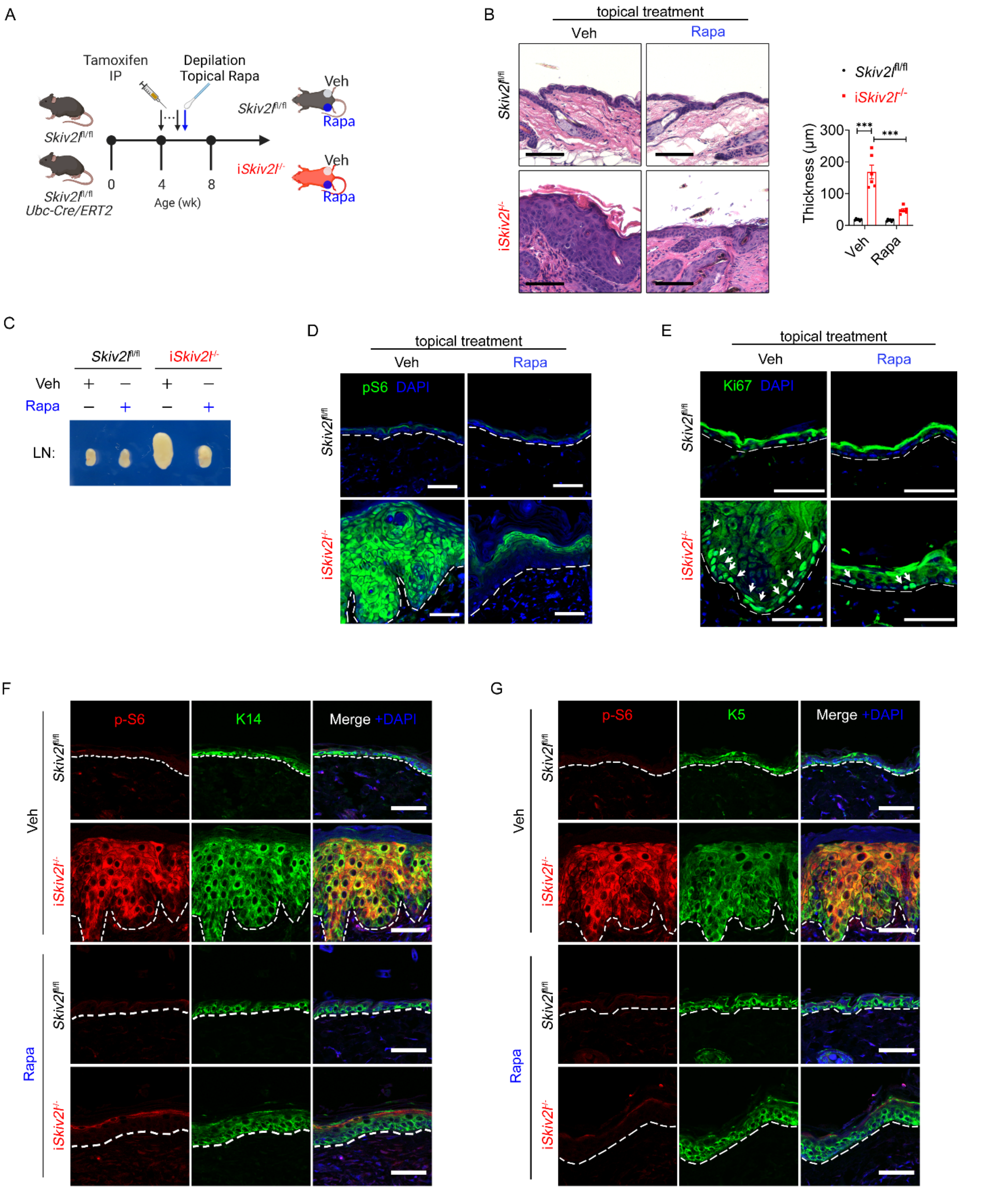

Supplemental Figure 12. Topical rapamycin treatment ameliorates skin pathology of postnatal whole-body inducible *Skiv2l* knockout mice

(**A**) A schematic diagram of experimental design for topical rapamycin treatment of i*Skiv2l*$^{-/-}$ mice and *Skiv2l*$^{fl/fl}$ controls after tamoxifen injection and depilation.

(**B**) H&E staining of i*Skiv2l*$^{-/-}$ and *Skiv2l*$^{fl/fl}$ mice skin topically treated with rapamycin or vehicle as above (**A**). Scale bar, 50 μm. Quantification of epidermal thickness is shown on the right bar graph. n=6 mice per group. Two-way ANOVA with post hoc Tukey's multiple comparisons test, ***P<0.001.

(**C**) Skin-draining inguinal and axillary lymph nodes of i*Skiv2l*$^{-/-}$ and *Skiv2l*$^{fl/fl}$ mice skin topically treated with rapamycin or vehicle.

(**D, E**) Fluorescent immunohistochemistry analysis of phospho-S6 ribosomal protein (S235/236) (**D**) and proliferation marker Ki67 (**E**) in i*Skiv2l*$^{-/-}$ and *Skiv2l*$^{fl/fl}$ mouse skin treated with rapamycin or vehicle. White arrows in (**E**), Ki67 positive cells. Dashed line, epidermal-dermal junction. Scale bar, 50 μm.

(**F, G**) Fluorescent immunohistochemistry analysis of phospho-S6 ribosomal protein (S235/236) and keratinocyte maker K14 (**F**) or K5 (**G**) in i*Skiv2l*$^{-/-}$ and *Skiv2l*$^{fl/fl}$ mouse skin treated with rapamycin or vehicle. Dashed line, epidermal-dermal junction. Dashed line, epidermal-dermal junction. Scale bar, 50 μm.

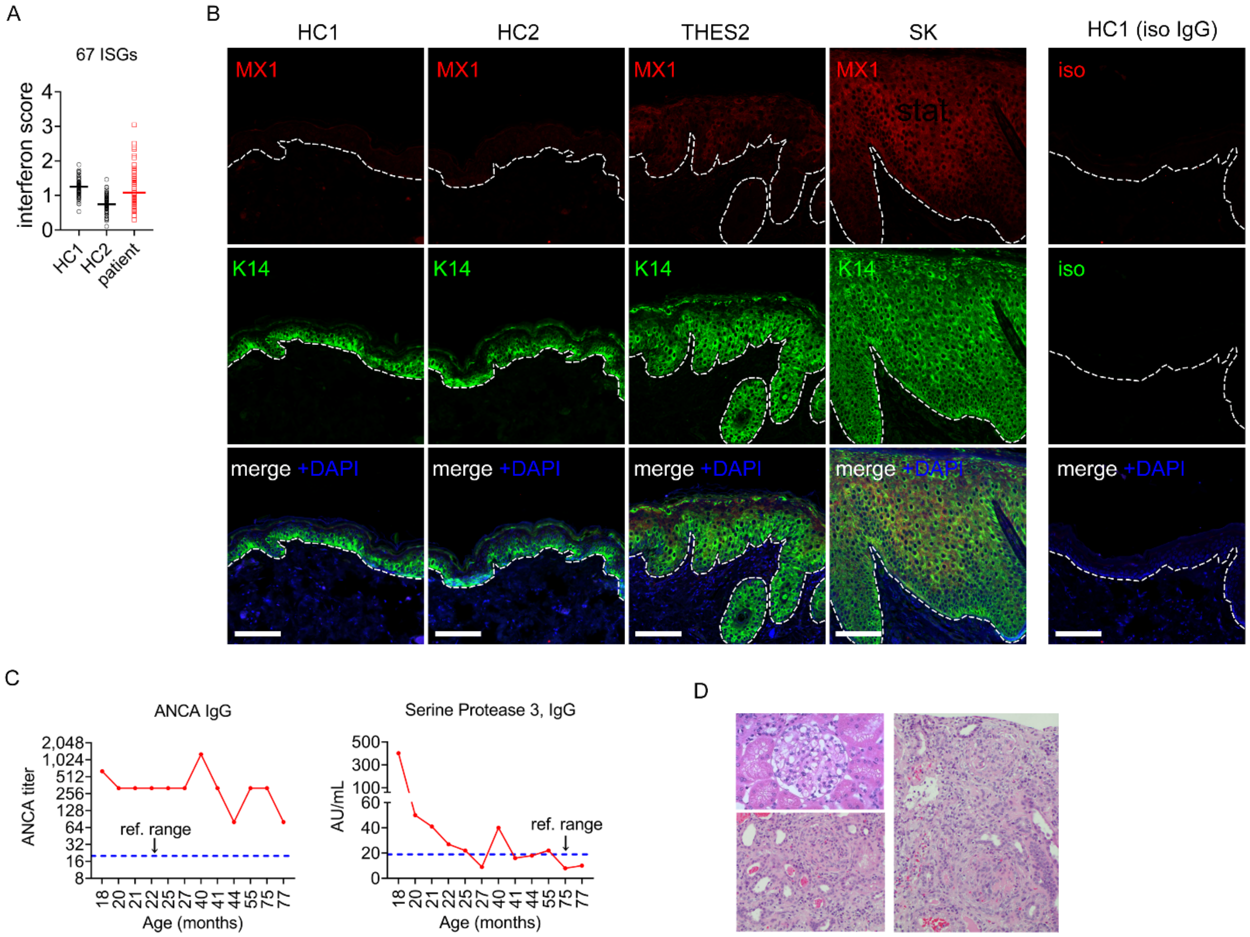

Supplemental Figure 13. Immunological parameters of the THES2 patient.

(**A**) Interferon score from average expression of 67 ISGs in PBMCs from the THES2 patient and two unrelated healthy controls (HC).

(**B**) Fluorescent immunohistochemistry analysis of MX1 and K14 in skin biopsies from healthy controls, THES2, and an unrelated case of seborrheic keratosis (SK). Dashed line, epidermal-dermal junction. Scale bar, 100 μm. The skin biopsy of THES2 is from the same patient as presented in **Figure 7E**.

(**C**) Titers of anti-neutrophil cytoplasmic antibody (ANCA) and serine protease 3 IgG antibodies in THES2 patient. Patient's titers are shown in solid red line and reference range are in dashed blue line.

(**D**) H&E staining of kidney biopsy at 17-month old indicates pauci-immune necrotizing and crescentic glomerulonephritis (NCGN), necrotizing arteriolitis. acute tubulointerstitial nephritis, global glomerulosclerosis, interstitial fibrosis and tubular atrophy.

Supplemental Table 1. qRT-PCR primer sequences

Gene	**Primer**	**Sequence 5'-3'**
Ifit1	Fwd	TTCACATGGAAGCTGCTATTTGAAA
	Rev	TGCTCAGCTGCTCGCTCTGGATCAA
Cxcl10	Fwd	GGGATCCCTCTCGCAAGGACGGTCC
	Rev	ACGCTTTCATTAAATTCTTGATGGT
Oas1a	Fwd	GGATGGCATAGATTCTGGGA
	Rev	CTGCATCAGGAGGTGGAGTT
Usp18	Fwd	GTGTCCGTGATCTGGTCCTT
	Rev	CTGCAGAAATACAACGTGCC
Rsad2	Fwd	GGACGCTTCATGGTGTTATTTG
	Rev	TGATTGGTCGCCTGTTTATCT
Ifnb	Fwd	CTGCGTTCCTGCTGTGCTTCTCCA
	Rev	TTCTCCGTCATCTCCATAGGGATC
Tnfa	Fwd	CTACCTTGTTGCCTCCTCTTT
	Rev	GAGCAGAGGTTCAGTGATGTAG
Il1b	Fwd	GGTACATCAGCACCTCACAA
	Rev	TTAGAAACAGTCCAGCCCATAC
Il6	Fwd	CACAAGTCCGGAGAGGAGAC
	Rev	CAGAATTGCCATTGCACAAC
Cxcl1	Fwd	CGAAGTCATAGCCACACTCAA
	Rev	GAGCAGTCTGTCTTCTTTCTCC
Cxcl2	Fwd	TAAGCACCGAGGAGAGTAGAA
	Rev	GTCCAAGGGTTACTCACAACA
Lce3a	Fwd	GTCTTGGGCTCTGTGTTCTT
	Rev	CATGGTTGGACACAGGTGAT
Rptn	Fwd	GAAGGAACACGGAGCCATAAA
	Rev	CCTTCAGACTGATTGTGGTGAG
Krt6a	Fwd	CAACATCATAACCCTCCCTGTC
	Rev	GAGGAAGCCAAGAGCATCAA
Krt6b	Fwd	GTCTCTGAGTTGCCTGGTAAAG
	Rev	CCAGGCCATTGGAAACTAGAA
Krt16	Fwd	CTCCTCTGGACAGTCCTATTCT
	Rev	GTCCCTGGAACTCTGACTTTG
Krt14	Fwd	GAGCGGCAAGAGTGAGATTT
	Rev	CTTTGGTCTCCTCCAGGTTATTC
Krt77	Fwd	GCTGCTTTCATGGGCAAATC
	Rev	GGGACAGCTCCGTATCAAATAG
Sprr2i	Fwd	TCCACATAGCACCTCCTTCTA
	Rev	ATTCTCTGCAGGCCCTTTAC
Sprr1b	Fwd	CCATATACCAGGCTCATCCATC
	Rev	GGCTGTTTCACTTGTTGCTC

S100a8	Fwd	CTTTGTCAGCTCCGTCTTCA
	Rev	TGTAGAGGGCATGGTGATTTC
S100a9	Fwd	GAGGAGTGTATGATGCTGATGG
	Rev	GTCACATGGCTGACCTCTTAAT
Defb4	Fwd	CGAAGAACCAGCAAGATGAATAAA
	Rev	CTAGAACTGGAGTTAGAGAAGGTAATC
Il33	Fwd	GGGTACCAAGCATGAAGAGAA
	Rev	GTCAACAGACGCAGCAAATG
Tslp	Fwd	TCATGACCTGACTGGAGATTTG
	Rev	AGCCAGGGATAGGATTGAGA
Slpi	Fwd	CGCCTCCTGGTAAAGACATAAA
	Rev	GGAGCACCGTGAAAGGTAAA
Klk6	Fwd	AGAGCACAGAACACTGCTAAAT
	Rev	CATCTGCTAACCACACCCATAG
Fabp4	Fwd	GCTCCTCCTCGAAGGTTTAC
	Rev	CCCACTCCCACTTCTTTCAT
Bcat1	Fwd	GTGGTAGCATTCGTGGGTAATA
	Rev	TCCTAAGCGCTGGGATTAAAG
Slc7a11	Fwd	GGGAAGGTGATGGCTGTATTT
	Rev	CACCACTCAGCATCTGTGTATC
Slc6a6	Fwd	CTGCCAAGTGACCCTCTAATC
	Rev	TAACACGAGAGCAACCAGAAATA
Cth	Fwd	GGTCCGGATGGAGAAACATT
	Rev	AGAGGGTAGCCCAGGATAAA
Ak4	Fwd	CCCTTCACAGGAACGAGTATAG
	Rev	CCTGTGGAAGGTGGGATAAA
Egln3	Fwd	GCCCAGGACTGCTTCTTATT
	Rev	TGGCATCTGTCACCAACTTTA
Slc2a1	Fwd	AAGACTGCTGCTCAGATCTATTC
	Rev	GGAGATAGGAGAGTGGCTGATA
Trib3	Fwd	GTCTCAGCTTTCTCCTGACTTT
	Rev	CACTAGGCACAGGAACGAATAA
Hk2	Fwd	CTCGGCCTTGTGAGTAGATAGA
	Rev	CGCGGGTGAATGAGGTATTT
Bhlhe40	Fwd	CTGCTGCCTTGCTTTCTTTC
	Rev	CTGAATCTTCTCTGTGGGTCTG
Mthfd2	Fwd	GTGCTTGGACCAGTACTCTATG
	Rev	CCAGCCACTACCACATTCTT
Eno1	Fwd	GATGGACGGCACAGAGAATAAA
	Rev	TCAGCAATGTGGCGGTAAA
Asns	Fwd	CAGTGTCTGAGTGCGATGAA
	Rev	CAAGCGGTGAAAGCCAAAG

Actb	Fwd	GAGGTATCCTGACCCTGAAGTA
	Rev	CACACGCAGCTCATTGTAGA
